# Untargeted Metabolomics Analysis Using FTIR and UHPLC-Q-Orbitrap HRMS of Two *Curculigo* Species and Evaluation of Their Antioxidant and α-Glucosidase Inhibitory Activities

**DOI:** 10.3390/metabo11010042

**Published:** 2021-01-08

**Authors:** Abdul Halim Umar, Diah Ratnadewi, Mohamad Rafi, Yohana Caecilia Sulistyaningsih

**Affiliations:** 1 Department of Biology, Faculty of Mathematics and Natural Sciences, Graduate School of Plant Biology, Jalan Agatis-Dramaga Campus, IPB University, Bogor 16680, Indonesia; ahuhalim76@gmail.com; 2 College of Pharmaceutical Sciences Makassar, STIFA Makassar, Jalan Perintis Kemerdekaan Km. 13.7 Daya, Makassar 90242, Indonesia; 3 Department of Biology, Faculty of Mathematics and Natural Sciences, Jalan Agatis-Dramaga Campus, IPB University, Bogor 16680, Indonesia; dratnadewi@apps.ipb.ac.id (D.R.); yohanasu@apps.ipb.ac.id (Y.C.S.); 4 Department of Chemistry, Faculty of Mathematics and Natural Sciences, Jalan Tanjung-Dramaga Campus, IPB University, Bogor 16680, Indonesia; 5 Advance Research Laboratory, Institute of Research and Community Services, Jalan Palem-Dramaga Campus, IPB University, Bogor 16680, Indonesia

**Keywords:** antioxidant activity, α-glucosidase inhibitor, *Curculigo* spp., FTIR, metabolomics, UHPLC-Q-Orbitrap HRMS

## Abstract

*Curculigo orchioides* and *C. latifolia* have been used as traditional medicines such as antidiabetic and anticancer. This study measured the total phenolics and flavonoid contents as well as analyzed the functional groups and chemical compounds using Fourier-transform infrared (FTIR) spectra and UHPLC-Q-Orbitrap-HRMS profiling for the discrimination of plant parts, geographical origin, and compounds that presumably have a significant contribution as antioxidant and α-glucosidase inhibitors on both plants. The total phenolics and flavonoids contents in *Curculigo* species varied from 142.09 to 452.47 mg gallic acid equivalent (GAE/g) and from 0.82 to 5.44 mg quercetin equivalent (QE/g), respectively. The lowest IC_50_ for antioxidant and α-glucosidase inhibitory activities is presented by *C. latifolia* from a higher altitude region. Principal component analysis (PCA) from FTIR and UHPLC-Q-Orbitrap-HRMS data could discriminate the plant parts and geographical origin. Partial least squares (PLS) analysis has identified several functional groups, such as O–H, C–H, C=O, C–C, C–O, and chemical compounds, unknown-185 and unknown-85, that are most likely to contribute to the antioxidant and α-glucosidase inhibitory activities.

## 1. Introduction

*Curculigo orchioides* Gaertn. and *Curculigo latifolia* Dryand. ex W.T.Aiton are widely distributed in Asia’s tropical and subtropical regions, mainly India, China, Indonesia, Malaysia, and Singapore. In Indonesia, the two *Curculigo* species are found from Sumatera to Papua. *Curculigo* spp. have several therapeutic properties because of their bioactive metabolites content. The rhizome of *Curculigo* species is traditionally used as estrogenic, neuroprotective, antibacterial, diuretics, aphrodisiac, antiapoptosis, anticancer, antidiabetic, and it neutralizes free radicals that likely could trigger various diseases. The leaves and flowers of *C. latifolia* are used to treat high fever, stomach-ache, and frequent urination [1,2]. The pharmacological effects of this species are attributed to the variety of their bioactive compounds, such as triterpene glycosides, phenolics, phenolic glycosides, and alkaloids.

A previous study [3] stated that the chemical components in *Curculigo* spp. possess some biological activities, such as cycloartane glycosides, which are used for leukemia treatment. Curculigosides F was found to inhibit hepatitis B virus (HBV) and antigen (HBeAg) in the HepG2.2.15 cell line, while chlorophenolic glycoside groups, such as curculigine M, curculigine N, and curculigine O, were reported to induce the proliferation of osteoblasts, orcinol glycosides, and 5,7,4′-trihydroxy-3′-methoxy flavone, which have antioxidant activity [4,5,6]. The phenolic group of *Curculigo* spp. is known as an antioxidant and has α-glucosidase inhibition activity (hydrolysis of α-1,6-glycosidic bonds) [7,8]. The metabolites in complex biological samples of *Curculigo* species could be identified rapidly and accurately using a metabolomics analysis to figure out their bioactive compounds. In the metabolomics analysis, there are two approaches to identifying the metabolites: the targeted and non-targeted analysis [9,10]. In the non-targeted analysis, metabolite fingerprinting and profiling could provide the complete identification of metabolites from a biological sample of *Curculigo* species. The analytical instrumentations used to study the metabolites of *Curculigo* species were Fourier-transform infrared (FTIR) spectrophotometer and UHPLC-Q-Orbitrap HRMS to obtain qualitative and quantitative data. Ultra-high performance liquid chromatography-mass spectrometry (UHPLC-MS) platforms have been widely used in plant science for metabolomics applications to identify and quantify compounds [10], and this approach is more sensitive and accurate for detecting metabolites [11].

It is not easy to find the correlation between FTIR spectra and UHPLC-Q-Orbitrap HRMS chromatograms with the contribution of biological activities related to its functional groups and metabolites due to the complexity of metabolomics data. Therefore, chemometrics analysis is needed in order to resolve this issue. The chemometric technique enables measurements of chemical systems through applications between mathematics and statistics, and it is an important method of analyzing much information from chromatographic data and can be used to evaluate the chemical similarities and dissimilarity of fingerprints between chromatographs of the sample [12]. Principal component analysis (PCA) and partial least squares (PLS) are the most frequent chemometrics analyses. PCA techniques make it possible to extract analytical information from chromatograms or spectra of samples to identify similarities and differences among highly complex datasets. PLS is one of the most popular calibration methods, which is based on determining the linear relationship between the independent dataset of measurement X (such as peak, absorbance values, fingerprint, spectra, or peak area from the detected metabolites) and the dependent set of variable Y (biological activity) in order to predict the bioactive compounds [13,14,15]. For the first time, we have performed metabolomics analysis using FTIR and UHPLC-Q-Orbitrap HRMS to identify major functional groups and metabolites in the two *Curculigo* spp. with a focus on substances having antioxidant and α-glucosidase inhibitory activities. We also differentiate the two *Curculigo* species based on their plant parts and their origin of geographical location.

## 2. Results

We designed the experiment based on the code samples, designating the species, the plant parts, and geographical location origin, which are summarized in Table 1. The highest total phenolics content was found in the rhizome of *C. latifolia* originated from Sinjai-Puncak (RLSK) (452.47 ± 0.12 mg GAE/g). In contrast, the lowest content of total phenolic contents was found in the leaves of *C. latifolia* from Sinjai-Biji Nangka (LLSB) (142.09 ± 0.63 mg GAE/g). The rhizome of *C. latifolia* harvested from different regions (RLSB and RLSP) also presented high total phenolics content, while *C. orchioides* rhizomes, regardless of their region, contained low phenolic content. In both species, phenolic compounds are accumulated more in the rhizomes than in the leaves and petiole of plant parts (Table 2).

The concentrations of total flavonoid in *C. latifolia* and *C. orchioides* are shown in Table 2. According to the plant parts and their origin location, the flavonoid concentrations in these two species are also varied. We found that the highest total flavonoid content was obtained in *C. orchioides* leaves (LOGM) as 5.44 ± 0.01 mg QE/g, while the lowest content was from *C. orchioides* rhizome (ROGM) as 0.82 ± 0.01 mg QE/g. Both samples were derived from the Gowa-Malakaji area in the southern part of Sulawesi island. The abundance of flavonoid compounds is more accumulated in the leaves than in the other plant parts (rhizome and/or petiole) of the two species.

The rhizome has a relatively higher antioxidant activity than the leaves and petiole of both species (Table 2), with the IC_50_ values varying between 47.08 and 473.04 mg/mL. *C. latifolia* rhizome has demonstrated the highest antioxidant activity, whereas the leaves of *C. orchioides* shows the second-highest antioxidant activity. The inhibitory concentration of α-glucosidase inhibitor from separated parts of the plant shows a different pattern with the values of IC_50_ varying from 155.25 to 553.38 mg/mL. The *C. latifolia* extract from the rhizome and leaves parts showed the highest α-glucosidase inhibitory activity among the other extracts from Sinjai-Puncak (RLSK and LLSK). Meanwhile, the *C. orchioides* extract from leaves and rhizome parts showed a weak effect as an inhibitor for α-glucosidase activity.

All samples of *Curculigo* species used in this study showed a specific FTIR spectrum profile, especially in the fingerprint region. The FTIR spectra derived from the ethanol extract of plant parts with the highest phenolic content are shown in Figure S1. At 3300 cm^–1^, the broad absorption bands indicated a stretching vibration from the O–H group. All samples of *Curculigo* species show the broad absorption band at a similar wavenumber. A stretching vibration at 3000 cm^−1^ was identified as C–H (sp^2^), which corresponds to the benzene ring, and a similar band was detected in all samples. However, the intensity of the absorption peak at 3000 cm^−1^ from the leaves of *C. orchioides* (LOBM), leaves of *C. latifolia* (LLSP), and petiole of *C. latifolia* (PLSP) samples was higher than that of the rhizome of *C. orchioides* (ROBM) and the rhizome of *C. latifolia* (RLSP) samples. The absorption band at 2800 cm^−1^ was related to the stretching vibration of C–H (alkane), and it was only observed in LOBM, LLSP, and PLSP samples. In the area of fingerprint wavenumber (1800–400 cm^−1^), only the absorption band in the range of 1152–1000 cm^−1^ was considered as C–O bending vibration. The absorption band in this range (1152–1000 cm^−1^) was detected in all samples, but the rhizomes of both *Curculigo* species manifested the highest intensity.

A typical chromatogram of UHPLC-Q-Orbitrap HRMS from both *Curculigo* species is shown in Figure S2, and it indicates abundant chemical compounds in their respective plant parts. This chromatogram profile shows a distinct variation in the retention times of 0.50–3.50, 14.00–16.00, and 23.00–27.00 min among parts of samples, i.e., leaves, petiole, and rhizome. The variation profile from sample ion chromatogram at these retention times can be distinguished in the number of peaks detected, peak height, and area.

We identified alkaloids, norlignan, phenolics, steroids, and terpenoids in *C.* *orchioides* and *C. latifolia* (Table S1). The compound identified from the triterpenoid (cycloartane) class in the *Curculigo* sample was curculigosaponin G, H, and I. Curculigosaponin G (*m/z* 783.488, ion mode [M + H]^+^) is fragmented into *m/z* 621.867 [M + H-C_6_H_12_O_5_]^+^, which indicates the release of C_6_H_12_O_5_ molecules followed by *m/z* 459.726 [M + H-C_6_H_12_O_5_]^+^ due to the release of C_6_H_12_O_5_ (Figure S3A). Curculigosaponin H (*m/z* 915.529, ion mode [M + H]^+^) is fragmented into *m/z* 751.350 [M + H-C_6_H_12_O_5_]^+^ and indicates the loss of C_6_H_12_O_5_ molecules followed by *m/z* 589.137 [M + H-C_6_H_12_O_5_]^+^ and 458.650 [M + H-C_5_H_10_O_4_]^+^, which indicates the release of C_6_H_12_O_5_ and C_5_H_10_O_4_ molecules (Figure S3B). Curculigosaponin I with *m/z* 945.150 in ion mode [M + H]^+^ fragmented into *m/z* 783.009, 621.867, and 458.726, which indicates the release of three C_6_H_12_O_5_ molecules (Figure S3C). The occurrence of fragmentations at *m/z* 459.726, 458.650, and 458.726 indicates the basic framework of triterpenoid saponins.

Curculigoside B (*m/z* 453. 136, ion mode [M + H]^+^) belongs to the phenolic compound class. It is fragmented into *m/z* 290.271 [M + H-C_6_H_12_O_5_]^+^ after releasing the C_6_H_12_O_5_ molecule and then becomes 276.244 [M + H-CH_2_]^+^ due to the release of CH_2_ (Figure S3D). Orchioside B (*m/z* 463.149, ion mode [M + H]^+^) is fragmented into *m/z* 350.143 [M + H-C_6_H_8_O_2_]^+^, which indicates the release of C_6_H_8_O_2_ molecules followed by *m/z* 213.143 [M + H-C_8_H_9_O_2_]^+^ due to the release of C_8_H_9_O_2_ molecules (Figure S3E).

One of the compounds identified from the norlignan class is (1S,2R)-O-Methylnyasicoside (*m/z* 493.167, ion mode [M + H]^+^). This compound is fragmented into *m/z* 475.159 and becomes 313.117 [M + H-C_6_H_12_O_5_]^+^ with the loss of C_6_H_12_O_5_ molecules (Figure S3F). The heatmap model (Figure S4A,B) depicts the variation of compounds found in the ion [M + H]^+^ and [M – H]^−^, which corresponds to its intensity from each plant part of both *Curculigo* species.

**Figure 1:**
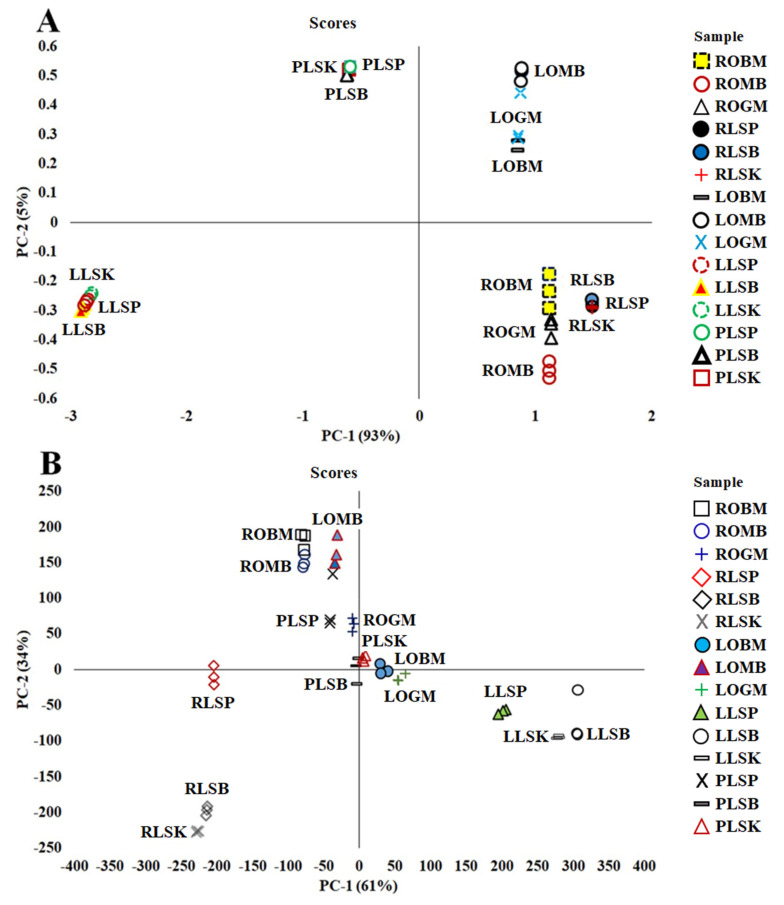
Principal component analysis (PCA) score plot for principal component (PC–1) and (PC–2) in 70% ethanol extract of *C. orchioides* and *C. latifolia* using Fourier-transform infrared (FTIR) spectra (**A**) and UHPLC-Q-Orbitrap HRMS (**B**).

Based on the PCA analysis shown (Figure 1A,B), each sample from the plant parts of the *Curculigo* species was divided into their respective group, namely, leaves, petiole, and rhizome, with a total variance value of the first two main components of 98% (PC-1, 93% and PC-2, 5%) and 95% (PC-1, 61% and PC-2, 34%).

**Figure 2:**
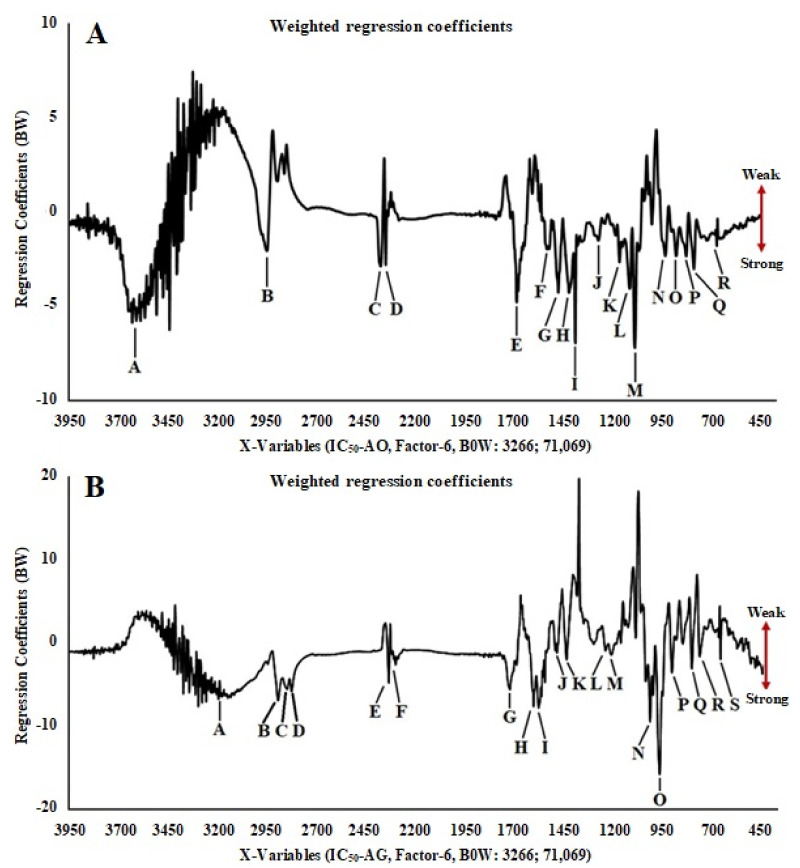
Partial least squares (PLS) plot weighted regression coefficients (important variables) of antioxidant (**A**) and α-glucosidase inhibitory (**B**) activities in 70% ethanol extract of *C. orchioides* and *C. latifolia* using FTIR spectra.

The PLS model was made from each spectral/chromatogram data variable (FTIR and UHPLC-Q-Orbitrap HRMS) and the biological activity assay. LV-1 and LV-2 values in the PLS model for antioxidant activity are 92% and 3%, and for α-glucosidase inhibition, they are 90% and 6%, respectively. The negative value of the PLS U score (factor-1) in the x-y relation outliers plot shows a strong contribution of each sample to the antioxidant (A) and α-glucosidase inhibition (B) activities (Figure S5). Line loading plots (Figure 2A,B) provided information on the functional groups and metabolites that contribute a major part to antioxidant activity and the inhibition of the α-glucosidase. Table 3 shows the wavenumbers, functional groups, and vibration modes present in all samples. The arrow mark at the right position of the line loading plots in Figure 2 indicates the level of bioactivity of *Curculigo* samples. The peaks at M (1083.920 cm^−1^) and I (1384.795 cm^−1^) with negative values and regression coefficients (BW) are −7.23 × 10^4^ and −7.90 × 10^4^, respectively. These peaks indicating the stretching vibrations of the C–O group (OH, COOH) and the bending vibrations of the O–H (phenol) group could have a major contribution to antioxidant activity. Meanwhile, the peaks in the area of O (975.914 cm^–1^) and N (1024.131 cm^–1^) with the smallest regression coefficients (BW) −5.91 × 10^4^ and −3.52 × 10^4^, respectively, indicate the stretching vibrations of the C–O group (trans disubstituent) and the bending vibrations of the C–H (alkenes), as well as the bending vibrations of the C–C group (cyclohexane). The three latter groups might have a major contribution to the inhibition of the α-glucosidase.

**Figure 3:**
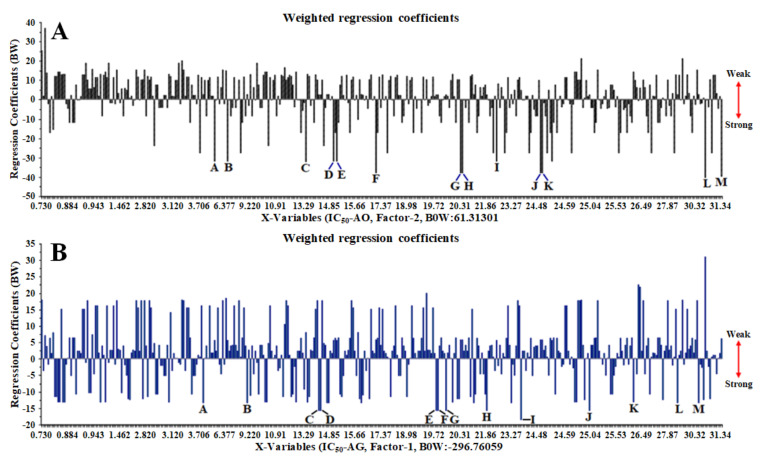
Partial least squares (PLS) plot weighted regression coefficients (important variables) of antioxidant (**A**) and α-glucosidase inhibitory (**B**) activities in 70% ethanol extract of *C. orchioides* and *C. latifolia* using UHPLC-Q-Orbitrap HRMS.

The chemical compounds, such as unknown-185 at a retention time of 30.920 min (peak L) and unknown-85 at 23.900 min (peak I), represent a strong (negative) signal to the antioxidant activity and α-glucosidase inhibition (Figure 3A,B). These chemical compounds predicted having a significant contribution to antioxidant activity and the inhibition of α-glucosidase, which are accumulated the highest in LLSB and LOBM samples and are all found in the leaves of *C. latifolia* and *C. orchioides*, respectively.

The FTIR and PLS analysis from *C. latifolia* rhizome can be obtained qualitatively, the functional groups of –OH, C=O, C–O, –C–H, C–C, and C–OH belong to these two triterpene compounds. These triterpene compounds are the major groups that might contribute the most to the antioxidant activity and α-glucosidase inhibition. Table 4 gives information about the retention times and BW values of these triterpene compounds. Their functional groups from the identified compounds may contribute to the antioxidant activity and the inhibition of α-glucosidase, which are summarized in Figure S6.

## 3. Discussion

The total phenolic and flavonoid contents in *C. latifolia* and *C. orchioides* varied depend on their harvest time, the origin of growth location, and the plant parts. The total phenolics compounds are all accumulated in the rhizomes part of plants. In this study, the total phenolic and flavonoid contents in RLSP, RLSB, and RLSK of *C. latifolia* samples are affected by its altitude. Its soil nutrient condition might influence the phenolic content in all *C. latifolia* samples, the soil moisture (microclimate), and the harvest time. RLSB and RLSK samples were harvested during the monsoon season and contained higher phenolic compound contents than the RLSP sample, which was harvested during the drought season. Oxidative stress caused by excessive or low soil water availability and unfavorable temperature can induce phenolic compounds’ synthesis for the plant survival [16,17].

The highest total flavonoid compounds are found in *C. orchioides*’ leaves and the leaves and petiole of *C. latifolia*. Flavonoids are accumulated in the aerial part of plants, especially leaves, and serve as phytoalexin to protect against pathogenic attacks and solar radiation, particularly ultraviolet radiation [18].

Some compounds are present in higher concentrations when certain exogenous effects are present. At higher altitudes, soil pH and soil micronutrient content decrease, which can be attributed to lower mineralization processes at lower pH. Some compounds (phenols, flavonoids, and terpenoids) increase in response to different altitudes. The accumulation of secondary metabolites is reported to have a vital role in tolerance to environmental biotic and abiotic stresses. These secondary metabolite help in the adaptation of stressed plants to different environmental conditions, for example, the accumulation of phenols and flavonoids as antioxidants [19].

Phenolic compounds prevent the enzymatic hydrolysis of carbohydrates by inhibiting α-amylase and α-glucosidase, with a mixed mechanism (competitive and uncompetitive) [20]. Previous studies using in vitro and in vivo testing [21,22,23] demonstrated an antidiabetic activity from the rhizome extracts of *C. orchioides* and *C. latifolia*. However, in this work, we found that only the rhizome of *C. latifolia* showed high antioxidant activity and potent inhibition of α-glucosidase enzyme associated with the high phenolic compounds in plants. In general, high phenolic content, along with high flavonoids in the samples, strongly correlates with antioxidants and α-glucosidase inhibition activities [24,25]. In our studies, the result is not in agreement with previous reports due to antioxidant and α-glucosidase inhibitory activity related to the other compounds, such as terpenoids, steroids, or alkaloids [26,27].

The FTIR spectra derived from *Curculigo* species show a distinct difference even though both plants are from the same genus. This is associated with both species having differences in the compound composition and content level of metabolites. The functional groups obtained from both extracts of *Curculigo* species are C–O, C=C, –CH, –COOH, and –OH, which are likely to be associated with antioxidant activity and α-glucosidase inhibitor. This assumption has been reported and described by Saleh et al. [28].

UHPLC-Q-Orbitrap HRMS can be operated with complete automatization with a short analysis time, and the extracted sample does not need further derivatization. The heatmap model was used to analyze each metabolite’s area from the *Curculigo* species’ extract and compare it for a similar compound. The heatmap model’s color intensity was used to depict the relative abundance in the sample extract of *Curculigo* species, and its variation can tell us about the compounds diversity in the plant [29]. We used the peak area from the detected metabolites because it is widely accepted that the peak area of an analyte in a chromatogram is directly proportional to its concentration [30].

*C. orchioides* has been reported to have an antioxidant activity associated with its compounds such as orcinol glycosides, curculigoside, curculigoside B, curculigoside C, curculigosaponin, 2,6-dimethoxyl benzoic acid, vanillin, and syringic acid [31,32]. A similar compound is also found in *C. latifolia*. Vanillin, which is phenolic acids, is used as a standard in several antioxidant studies. Both types of phenolic acids have more significant antioxidant activity than gallic acid [33]. At the same time, lycorine has also been determined in the rhizome and leaves of both *Curculigo* species. This compound, a type of alkaloid widely found in the genus Amaryllidaceae, has been reported before having an antioxidant and α-glucosidase inhibition activity [34].

In this study, PCA, as an unsupervised pattern recognition technique, has been used for obtaining the information of patterns of sample grouping, similarities, and differences among species and plant parts [35,36]. A PCA plot will consist of two principal components (PCs) because the first two PCs capture most of the variant of the data the closer the PC values of the samples. The cumulative percentages of total variance (PC-1 and PC-2) obtained from FTIR spectra and UHPLC-Q-Orbitrap HRMS data were 98% and 95%, respectively. It can be concluded that the PCA analysis used for sample clustering shows good accuracy in both terms of species and plant parts.

PLS, as a supervised pattern recognition method, is often used to correlate medicinal plants’ biological activities with chromatographic or spectroscopic data (peak area or peak and absorbance value) [15,30,37]. The parameters used to create the PLS model are latent variables (LV), and its full cross-validation was applied for data validation and avoiding overfitting [38,39]. As suggested by Easmin et al. [40], line loading plots (weighted regression coefficients) in the PLS model were used to evaluate functional groups and chemical compounds, and also for samples clustering having low or high activity (x-y relation outliers) from the FTIR spectrum and UHPLC-Q-Orbitrap HRMS data. Based on the results from PLS analysis, O–H, C–H, C=O, C–O, and C–C functional groups in the chemical structure of unknown-185 and unknown-85 are highly observed in the LLSB of *C. latifolia* and LOBM samples of *C. orchioides.* This is predicted to significantly contribute to the antioxidant activity and α-glucosidase inhibition, whereas the phenolics (curculigoside B, 2,4-dichloro-5-methoxy-3-methylphenol, orcinol glucoside, and orchioside B), cycloartane triterpene (curculigosaponin G, H, and I), and norlignan compounds (1,1-Bis(3,4-dihydroxyphenyl)-1-(2-furan)-methane and (1S,2R)-O-Methylnyasicoside) have an only minor contribution to the biological activities.

Interestingly, from this study, the active compounds are not always derived from the most abundant compounds found in the extract of *Curculigo* species. Other functional groups are also present in the chemical compounds from cycloartane triterpene (curculigosaponin G, H, and I), i.e., hydroxyl groups at the positions C–3 and C–11, and a double bond at C23=C24. It has been pointed out that the double bonds at C24=C25 of the tetracyclic triterpenoids have an essential role as antioxidant and α-glucosidase inhibitors [26,41].

## 4. Materials and Methods

### 4.1. Chemicals and Reagents

Alpha-glucosidase enzymes (≥98%), gallic acid (≥98%), quercetin (≥98%), p-nitrophenyl-α-D-glucopyranoside (≥99%), and 1,1-diphenyl-2-picrylhydrazyl (DPPH) (≥98%) were purchased from Sigma Aldrich (St Louis, USA). Water (LC-MS grade), acetonitrile (LC-MS grade ≥ 99.9%), methanol (LC-MS grade ≥ 99.9%), formic acid (≥98%), potassium bromide (KBr, FTIR grade), aluminum chloride (≥98%), sodium carbonate (≥98%), and DMSO (≥99.9%) were purchased from Merck (Darmstadt, Germany).

### 4.2. Plant Material

The plant samples from *Curculigo* species were collected during 2018–2019 from four regencies in Sulawesi Island, Indonesia: Barru, Maros, Gowa, and Sinjai. The coordinates, plant parts, sample code, collection time (season), and altitude are summarized in Table 1, and the geographical locations are shown in Figure S7. All samples were identified based on local knowledge and by comparing them to the published flora and voucher specimens stored at the Tropical Biopharmaca Research Center, IPB University, Indonesia.

### 4.3. Sample Preparation

The plant samples were air-dried for three days in a drying cabinet at 40 °C. Each sample (0.5 kg) was pulverized and sieved with a 40-mesh sieve. The samples were extracted by the maceration method using 5 L of ethanol (70%) for 24 h at room temperature. A similar procedure was repeated twice. Then, the sample was filtrated, which was followed by filtrate evaporation using a rotary evaporator (Rotavapor R-210, BÜCHII Labortechnik, Switzerland) below 50 °C until obtaining a dry condition.

### 4.4. Determination of Total Phenolics

The total phenolics content was determined using the method described by Margraf et al. [42] with slight modifications. Approximately a 20 µL sample was transferred to a 96-well microplate (Costar^®^, Washington, DC, USA), which was followed by the addition of 110 µL of Folin–Ciocalteu and 70 µL sodium carbonate (0.01 M). After vortexing, the sample mixture was incubated for 30 min at 37 °C. The absorbance was measured at 750 nm. Phenolics content was expressed as gallic acid equivalent (mg GAE/g) by using a calibration curve of gallic acid (y = 0.0091x − 0.0123, with R^2^ = 0.9982).

### 4.5. Determination of Total Flavonoids

A procedure from Zhu et al. [43] was used for the determination of total flavonoids with slight modifications. Briefly, 10 µL of the sample extract was mixed with 60 µL ethanol, 10 µL AlCl_3_ (10% *b*/*v*), 10 µL potassium acetate (1 M), and 120 µL distilled water. The mixture was added to a 96-well plate, incubated for 30 min at 37 °C, and the absorbance was read at 450 nm. The total flavonoid content was expressed as quercetin equivalent (mg QE/g) by using the calibration curve of quercetin (y = 0.003x − 0.0157, with R^2^ = 0.9935).

### 4.6. Antioxidant Activity by DPPH Method

We used the DPPH method for the measurement of radical scavenging activity, according to Ananthi et al. [44], with slight modifications. About 10 mg of each sample extract was dissolved in 1 mL of ethanol 70%. We added 100 µL of sample solution into a 96-well plate and then mixed it with 100 µL of DPPH 125 µM (in ethanol). The mixture was incubated at 37 °C for 30 min, and the absorbance was measured at 517 nm. The percentage of scavenging activity of the extract was calculated by using the following equation:

(1) $$\%\;\mathrm{Scavenging}\;\mathrm{activity}\;=\left(1\;-\;\frac{\left(A\;-\;B\right)}{\left(C\;-\;D\right)}\right)\;\times \;100\%$$

where *A* = absorbance of sample, *B* = absorbance of sample correction, *C* = absorbance of blank, and *D* = absorbance correction of the blank. Antioxidant activity was determined using the following equation: y = 0.06665x − 0.0333, with R^2^ = 0.9984. Quercetin was used as the standard.

### 4.7. α-Glucosidase Enzyme Inhibition Assay

The method for determining α-glucosidase enzyme inhibitory activity has been previously described by Hamid et al. [45] with slight modifications. The reaction mixture consisted of a 10 µL sample extract, 50 µL phosphate buffer (0.1 M; pH 7), 25 µL 4-nitrophenyl α-d-glucopyranoside, 0.01 M substrate in phosphate buffer (pH 7), and 25 µL α-glucosidase enzyme (0.1 U/mL α-glucosidase solution in 0.01 M phosphate buffer pH 6). The mixture was added to a 96-well plate, and the plate was agitated slowly for 5 min. After being homogenized, the mixture was incubated at 37 °C for 20 min. The reaction process was stopped by adding 100 µL of sodium carbonate. The α-glucosidase inhibitory activity was determined by measuring the absorbance at 410 nm in a microplate reader. The following equation was used for the determination of α-glucosidase inhibitory activity according to antioxidant activity.

The inhibitory activity was determined using the following equation: y = 13.516 ln(x) + 68.941, with R^2^ = 0.9272. Acarbose was used as the standard.

### 4.8. Measurement of FTIR Spectrum

Dried extracts (5 mg) of each sample were mixed with 95 mg of KBr and then compressed to form a tablet (3 mm) before the analysis by FTIR spectrophotometry. The FITR spectrophotometer (Tensor 37, Bruker Optik GmbH, Germany) was equipped with a DTGS (deuterated detectors triglycine sulfate). FTIR spectra were recorded in the region of 4000–400 cm^–1^, in absorbance mode, 32 scans/min, and resolution of 4 cm^–1^. The spectra were processed by OPUS ver. 4.2 software (Bruker, Germany) and Spectragryph v1.2.11. This analysis was used to obtain the spectrum pattern from the sample of plant parts, i.e., leaves, petiole, and rhizome. Then, the obtained spectrum was analyzed by chemometrics for the identification, discrimination, groupings, and detecting any functional groups that might contribute to the antioxidant activity and α-glucosidase inhibition.

### 4.9. Identification of Metabolites by UHPLC-Q-Orbitrap HRMS

This qualitative analysis referred to the method developed by He et al. [31], with various modifications. Approximately 50 mg of dried extract was ultrasonically extracted (Branson Ultrasonic Corporation, Danbury, CT, USA) with 1.5 mL of methanol at 30 °C for 15 min. Then, the mixture was filtered through a 0.2 µm syringe filter membrane (SY25TF PTFE mdi), and the filtrate was collected in a vial. Metabolite profiling was performed in a Vanquish Flex UHPLC-Q Exactive Plus Orbitrap High-Resolution Mass Spectrometer using Accucore^TM^ Phenyl Hexyl (100 × 2.1 mm, 2.6 µm) as the separation column and UV detector at 254 nm. The source of MS ionization used was electrospray ionization (ESI) and Q-Orbitrap was used as the mass analyzer. The collision energy used for fragmentation was 18, 35, and 53 eV. Other conditions were as follows: spray voltage 3.8 kV, a capillary temperature of about 320 °C, sheath gas and auxiliary gas flow rates of 15 and 3 mL/min, respectively.

The flow rate from the delivery system was adjusted at 0.3 mL/min, the autosampler temperature was maintained at 10 °C, and the sample injection volume was 0.5 µL. The mobile phase consisted of (A) 0.1% formic acid in water and (B) acetonitrile. A linear gradient elution program was applied as follows: 0–1.5 min (5% B), 1.5–9 min (5–10% B), 9–13 min (10–20% B), 13–17 min (20–28% B), 17–23 min (28–78% B), 23–26 min (70–95% B), 26–29 min (95% B), and 29–32 min (5% B). The total run time was 32 min, with relative abundance 0–100 and MS full-scan type (100–1500) in positive and negative mode.

### 4.10. Data Analysis

The experimental results of total phenolics and flavonoid contents and the determination of antioxidant and α-glucosidase inhibitory are expressed as means ± SD of three replicates for each sample. One-way ANOVA used R ver. I386 3.6.0 and significance levels were tested at *p* < 0.05 (Welch’ s test).

The processed data from FTIR (spectra data) [15] and UHPLC-Q-Orbitrap HRMS (peak area value) [46] were analyzed using the UnscramblerX ver. 10.4. (CAMO, Norwegia) for multivariate data analysis (MVDA). In order to access the information of patterns of sample grouping, similarities, and differences among species and plant parts, principal component analysis (PCA) was used. In this PCA method, data preprocessing uses a spectroscopic model, validation with full cross-validation, and an algorithm using singular value decomposition (SVD). The partial least squares (PLS) method was conducted to analyze further the correlation of antioxidant and α-glucosidase inhibitory activity with functional groups and the chemical compounds in the *Curculigo* spp. samples. In the PLS method, data preprocessing uses a spectroscopic model, validation with full cross-validation, and an algorithm using the Kernel PLS.

Qualitative analysis of the compounds was analyzed using the MS-DIAL ver. 3.82 (peak discrimination, filtering, and alignment). MS raw data (*.raw format) were imported to Abf Converter.4.0.0 (*.abf format) and MS FileReader 2.2.62. The peak detection parameters used are minimum peak height 10,000 (Orbitrap), smoothing method (linear weighted moving average), smoothing level (2), and minimum peak width (3). Identification used an in-house database with a score cut off (75%), adduct type [M + H]^+^ and [M − H]^−^, and alignment using reference blanks. Then, for the compounds identified, we calculated the mass error value (10 ppm) as the filtering criteria to scan experiments. Fragmentation patterns (MS and MS/MS) were analyzed using Compound Discoverer™ Software (Thermo Scientific™, Waltham, MA, USA), and there are freely available software fragmentation algorithms such as MS-FINDER, CFM-ID (https://cfmid.wishartlab.com/), MetFrag, and CSI:FingerID (https://www.csi-fingerid.uni-jena.de/).

In addition, a heatmap of hierarchical cluster analysis was conducted to present the results of putatively identified compounds in plant parts extracts of *Curculigo* spp. and can be used to discover clustering patterns in the datasets. Heatmap was constructed with MetaboAnalyst 4.0. (https://www.metaboanalyst.ca) using peak areas data (*.csv format) from all compounds detected in the adduct type ([M + H]^+^ and [M − H]^−^) of each organ of *Curculigo* species. For hierarchical clustering analysis, distance measures using Euclidean, clustering algorithms using complete, and standardization using autoscale were chosen [30,47].

## 5. Conclusions

*C. latifolia*’s rhizome contains a more significant amount of total phenolics than all parts of *C. orchioides*, and the phenolic compounds are more active as an antioxidant and α-glucosidase inhibitor than flavonoids. FTIR, UHPLC-Q-Orbitrap HRMS, and assays for biological activities, combined with PCA, can be used as a comprehensive method to distinguish the two species, *C. orchioides* and *C. latifolia*, and also their plant parts. Using PLS analysis, we can predict the functional groups and the chemical compounds contributing to antioxidant and α-glucosidase inhibitory activities. The chemical compounds that contribute to the antioxidant activity and the inhibition of α-glucosidase, unknown-185 and unknown-85, are accumulated the highest in LLSB and LOBM samples, and all are founded in leaves of *Curculigo* species. Based on FTIR and PLS analysis, –OH, C=O, C–O, –C–H, C–C, and C–OH groups from these compounds are the major groups that might contribute the most to the biological activities in *Curculigo* plant species.

## Figures and Tables

**Table 1:** Sources of plant samples in South Sulawesi.

Sample Species	Regency (District)	Coordinate	Organ	Sample Code	Collection Time	Season	Altitude (MASL)
*C. orchioides*	Barru (Mallawa)	4°12′00′′ S 19°38′31′′E	Rhizome Leaves	ROBM LOBM	July 2018	Dry	14
	Maros (Bengo-bengo)	5°00′09′′ S 19°45′56′′ E	Rhizome Leaves	ROMB LOMB	July 2018	Dry	462
	Gowa (Malakaji)	5°26′06′′ S 19°50′12′′ E	Rhizome Leaves	ROGM LOGM	July 2018	Dry	750
*C. latifolia*	Sinjai (Palangka)	5°18′04′′ S 20°06′33′′ E	Rhizome Leaves Petiole	RLSP LLSP PLSP	July 2018	Dry	483
	Sinjai (Biji Nangka)	5°18′43′′ S 120°05′25′′ E	Rhizome Leaves Petiole	RLSB LLSB PLSB	February 2019	Rainy	640
	Sinjai (Puncak)	5°13′25′′ S 120°02′46′′ E	Rhizome Leaves Petiole	RLSK LLSK PLSK	February 2019	Rainy	959

MASL: meters above sea level.

**Table 2:** Total phenolics, total flavonoids, antioxidant, and α-glucosidase inhibitory activities in 70% ethanol extract of *C. orchioides* and *C. latifolia*.

Sample Code	Total Phenolics (mgGAE/g)	Total Flavonoids (mgQE/g)	IC_50_ (mg/mL Extract)	
			Antioxidant (DPPH)	α-Glucosidase Inhibitory
ROBM	242.58 ± 0.49 ^e^	0.85 ± 0.01 ^a^	71.09 ± 2.25 ^b^	537.82 ± 6.87 ^h^
ROMB	288.20 ± 0.90 ^h^	0.89 ± 0.01 ^a^	102.93 ± 0.53 ^c^	522.00 ± 5.32 ^h^
ROGM	254.66 ± 0.44 ^f^	0.82 ± 0.01 ^a^	165.54 ± 1.26 ^e^	434.65 ± 5.55 ^fg^
RLSP	429.63 ± 0.42 ^l^	0.88 ± 0.02 ^a^	52.30 ± 0.07 ^a^	378.39 ± 8.17 ^cde^
RLSB	435.98 ± 0.32 ^m^	1.37 ± 0.01 ^b^	50.68 ± 0.41 ^a^	182.73 ± 3.87 ^a^
RLSK	452.47 ± 0.12 ^n^	1.57 ± 0.01 ^c^	47.08 ± 1.35 ^a^	155.25 ± 0.68 ^a^
LOBM	303.38 ± 0.95 ^j^	5.10 ± 0.01 ^h^	165.84 ± 0.57 ^e^	553.38 ± 12.22 ^h^
LOMB	170.90 ± 0.95 ^c^	2.72 ± 0.01 ^d^	161.44 ± 4.82 ^e^	349.14 ± 4.09 ^bd^
LOGM	194.22 ± 0.80 ^d^	5.44 ± 0.01 ^i^	205.89 ± 4.31 ^f^	349.93 ± 4.17 ^bd^
LLSP	168.95 ± 0.44 ^c^	4.21 ± 0.03 ^e^	363.12 ± 4.00 ^h^	325.45 ± 2.89 ^b^
LLSB	142.09 ± 0.63 ^a^	4.85 ± 0.01 ^g^	473.04 ± 0.12 ^j^	328.82 ± 21.48 ^bc^
LLSK	151,86 ± 1.16 ^b^	4.41 ± 0.01 ^f^	447.70 ± 0.76 ^i^	300.02 ± 0.97 ^b^
PLSP	278.23 ± 0.24 ^g^	4.39 ± 0.04 ^f^	140.45 ± 0.79 ^d^	462.72 ± 23.22 ^g^
PLSB	331.83 ± 0.42 ^k^	4.85 ± 0.01 ^g^	223.88 ± 0.91 ^g^	397.56 ± 11.19 ^df^
PLSK	229.35 ± 0.53 ^i^	4.18 ± 0.02 ^e^	213.23 ± 1.24 ^fg^	404.43 ± 2.53 ^ef^

The reported value is the mean ± SD of a triplicate assay for each sample. The values in the same column, followed with different superscript letters, indicate a significant difference at the level of *p* < 0.05 (Welch’s test).

**Table 3:** The results of partial least squares (PLS) analysis on the wavenumbers, functional group, and vibration mode contribute to the antioxidant and α-glucosidase inhibitory activities.

In Vitro Assay	Peak Code	Wavenumber (cm^−1^)	Functional Group	Vibration Mode
Antioxidant activity	A	3606.638	O–H	Stretching
	B	2945.009	C–H	Stretching
	C	2370.352	O=C=O, N–H	Stretching
	D	2345.2	O=C=O	Stretching
	E	1681.812	C=O	Stretching
	F	1527.517	C=C (aromatic)	Stretching
	G	1471.584	O–H	Bending
	H	1417.582	–C–H (CH_3_)	Bending
	I	1384.795	C–O	Bending
	J	1267.145	C–O (OH, COOH)	Bending
	K	1162.996	C–C	Bending
	L	1108.993	C–O	Stretching (aliphatic of ether)
	M	1083.92	C–O	Stretching (secondary alcohol)
	N	933.483	–C–H (alkene)	Bending (trans disubtituen)
	O	875.622	–C–H (aromatic)	Bending
	P	821.619	–C–H (aromatic)	Bending (trisubtituen)
	Q	784.974	C–Cl	Bending
	R	669.253	C–X (halogen)	Halogen and aromatic disubtituen
*α*-glucosidase inhibitory activity	A	3170.755	O–H	Stretching
	B	2916.169	C–H	Stretching
	C	2869.881	C–H	Stretching
	D	2842.879	C–H	Stretching
	E	2352.994	O=C=O	Stretching
	F	2318.277	N–H, O–H, S–H	Streching
	G	1737.744	C=O	Stretching
	H	1616.237	N–H (NH_2_)	Bending
			C=C (alkene)	Stretching
	I	1591.164	C=C (aromatic)	Streching
	J	1496.658	C=N, N=O	Bending
			C=C (alkene)	Stretching
	K	1448.441	–C–H (–CH_3_)	Bending
	L	1251.715	C–O	Stretching
	M	1220.857	C–O	Stretching
	N	1024.131	C–N	Stretching
	O	975.914	C–H	Bending
	P	914.196	C–N	Bending
	Q	811.976	C–H	Bending
	R	775.437	C–Cl	Stretching
	S	665.396	C–X (halogen)	Halogen and aromatic disubtituen

**Table 4:** The compounds identified in the 70% ethanol extracts of *C. orchioides* and *C. latifolia* using UHPLC-Q-Orbitrap HRMS that contributed to the antioxidant and α-glucosidase inhibitory activity.

Peak Code	RT [min]	BW	Type	Metabolite Name	Formula	Chemical Type
Antioxidant Activity						
A	5.945	−31.8958	[M + H]^+^	Curculigoside B	C_21_H_24_O_11_	Phenolic glycosides
B	6.680	−31.8958	[M + H]^+^	Unknown-173	C_6_H_10_O_13_	-
C	13.320	−32.1579	[M − H]^−^	Unknown-99	C_37_H_39_N_14_O_20_	-
D	14.930	−31.8958	[M + H]^+^	Unknown-176	C_15_H_13_O_8_	-
E	15.050	−31.8958	[M − H]^−^	Unknown-175	C_26_H_35_O_16_	-
F	16.848	−37.8736	[M + H]^+^	(1S,2R)-O-Methylnyasicoside	C_24_H_28_O_11_	Norlignan
G	20.320	−37.8736	[M − H]^−^	1,1-Bis(3,4-dihydroxyphenyl-1-(2-furan)-methane	C_17_H_14_O_5_	Norlignan
H	20.320	−37.8736	[M − H]^−^	Curculigosaponin G	C_18_H_18_O_6_	Cycloartane (Triterpene)
I	22.410	−31.8958	[M + H]^+^	Unknown-179	C_47_H_49_O	-
J	24.506	−37.8736	[M − H]^−^	Curculigoside B	C_21_H_24_O_11_	Phenolic glycosides
K	24.506	−37.8736	[M − H]^−^	Orchioside B	C_23_H_26_O_10_	Phenolic glycosides
L	30.920	−40.3125	[M + H]^+^	Unknown-185	C_47_H_59_O_7_	-
M	31.435	−39.7257	[M − H]^−^	2,4-Dichloro-5-methoxy-3-methylphenol	C_8_H_8_Cl_2_O_2_	Phenolic
α-Glucosidase inhibitory						
A	4.060	−13.3962	[M + H]^+^	Unknown-76	C_13_H_9_O_11_	-
B	8.770	−13.3962	[M − H]^−^	Unknown-77	C_13_H_30_N_5_O_11_	-
C	14.272	−15.7379	[M + H]^+^	Orcinol glucoside	C_13_H_18_O_7_	Phenolic glycosides
D	14.272	−15.7379	[M + H]^+^	1,1-Bis(3,4-dihydroxyphenyl-1-(2-furan)-methane	C_17_H_14_O_5_	Phenolic
E	19.801	−15.7379	[M + H]^+^	5-Hydroxymethylfurfural	C_6_H_6_O_3_	Aldehyde
F	19.801	−15.7379	[M + H]^+^	Curculigosaponin G	C_42_H_70_O_13_	Cycloartane (Triterpene)
G	20.049	−15.7379	[M + H]^+^	Curculigosaponin I	C_48_H_80_O_18_	Cycloartane (Triterpene)
H	22.200	−15.7379	[M − H]^−^	Unknown-84	C_30_H_61_O_19_	-
I	23.900	−18.5262	[M + H]^+^	Unknown-85	C_42_H_51_O_6_	-
J	25.040	−15.7379	[M + H]^+^	Unknown-87	C_57_H_86_N_5_O_5_	-
K	26.030	−13.1626	[M − H]^−^	Unknown-10	C_7_H_12_NO_12_	-
L	29.010	−13.3962	[M + H]^+^	Curculigosaponin H	C_47_H_78_O_17_	Cycloartane (Triterpene)
M	30.901	−13.3962	[M − H]^−^	Unknown-88	C_54_H_68_O_2_	-

## Data Availability

The data presented in this study are available in https://www.mdpi.com/2218-1989/11/1/42/s1.

## Supplementary Materials

The following are available online at https://www.mdpi.com/2218-1989/11/1/42/s1. Figure S1: FTIR spectra of a 70% ethanol extract of *C. orchioides* and *C. latifolia* plant parts, Figure S2: Typical total ion chromatograms profiles of 70% ethanol extracts of *C. orchioides* and *C. latifolia* plant parts, Figure S3: The proposed fragmentation patterns of representative curculigosaponin G (triterpenoid) (**A**), curculigosaponin H (triterpenoid) (**B**), curculigosaponin I (triterpenoid) (**C**), curculigoside B (phenolic) (**D**), orchioiside B (phenolic) (**E**), and (1S,2R)-O-Methylnyasicoside (norlignan) (**F**) at ion mode [M + H]^+^) and [M − H]^−^) in *C. orchioides* and *C. latifolia*, Figure S4: Heatmap correlation between chemical compounds in 70% ethanol extract of *C. orchioides* and *C. latifolia* and their relative abundance. Positive mode (**A**) and negative mode (**B**). The color scale differentiates values: high (red), moderate (black), and low (green), Figure S5: Partial least squares (PLS) plots x-y relation outliers antioxidant (**A**) and α-glucosidase inhibitory (**B**) activity of a 70% ethanol extract of *C. orchioides* and *C. latifolia*. Axis and ordinate plots show PLS scores for each organ sample, Figure S6: The chemical structure of curculigosaponin G (**A**), curculigosaponin H (**B**), curculigosaponin I (**C**), curculigoside B (**D**), orchioiside B (**E**), and (1S,2R)-O-Methylnyasicoside (**F**) and their functional groups might have a major contribution to the antioxidant activity and the inhibition of α-glucosidase, Figure S7: Sampling location of *Curculigo* spp. in South Sulawesi, Sulawesi Island, Indonesia. *Curculigo orchioides* Gaertn. (**A**) and *Curculigo latifolia* Dryand. ex W.T.Aiton (**B**), Table S1: The chemical compounds identified in all sample extracts of *C. orchioides* and *C. Latifolia*.
