# Supplementary material for: Untargeted Metabolomics Analysis Using FTIR and UHPLC-Q-Orbitrap HRMS of Two Curculigo Species and Evaluation of Their Antioxidant and α-Glucosidase Inhibitory Activities
Source: Metabolites. 2021 Jan 8;11(1):42. doi: 10.3390/metabo11010042 (PMC7827591; doi:10.3390/metabo11010042)
Supplement: Supplementary file 1 [file metabolites-11-00042-s001.pdf]

Supplementary Material

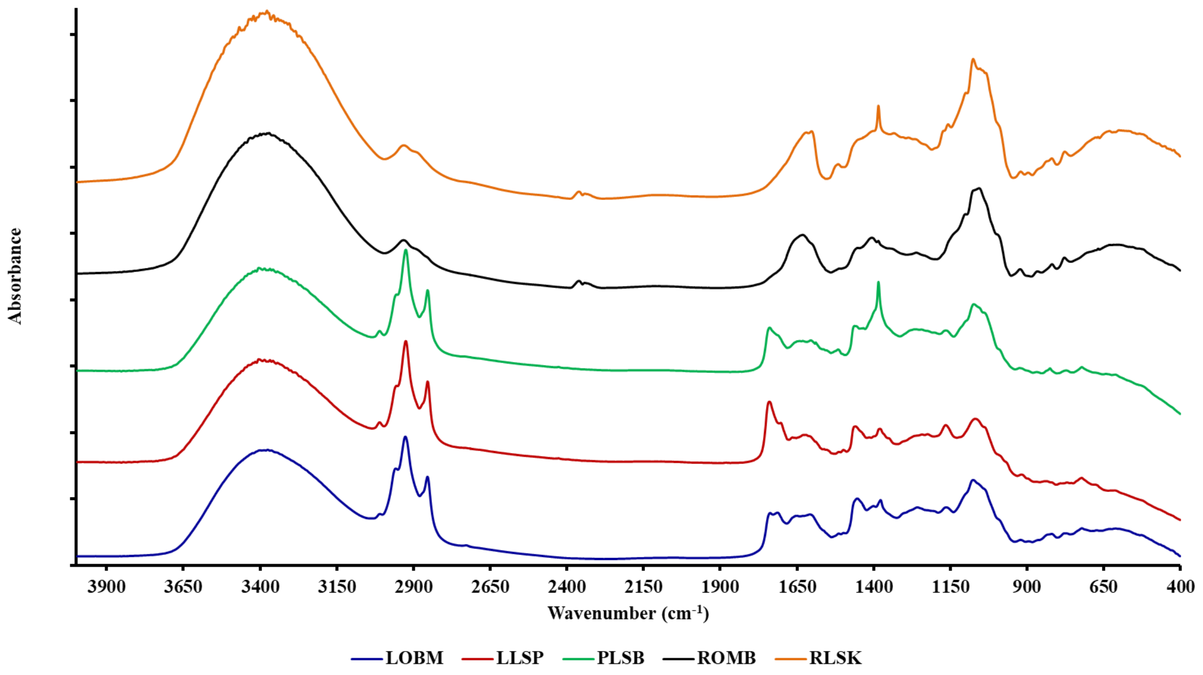

Figure S1. FTIR spectra of a 70% ethanol extract of *C. orchioides* and *C. latifolia* plant parts.

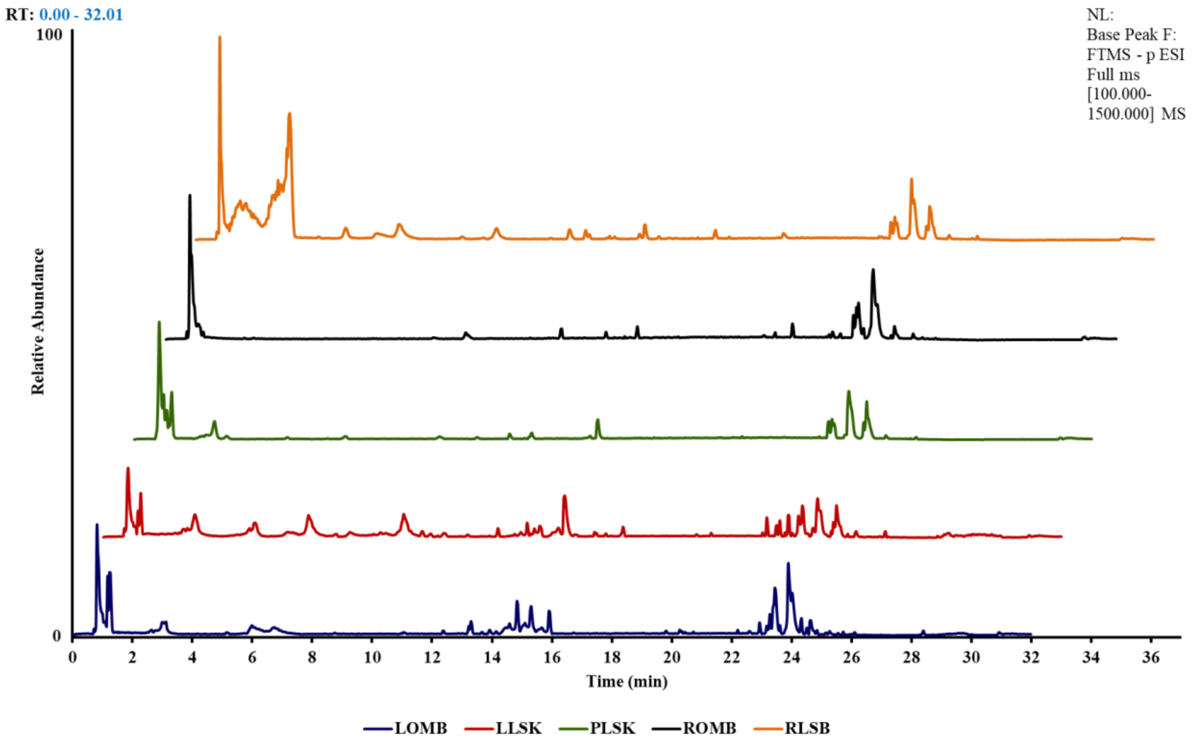

Figure S2. A typical total ion chromatograms profiles of 70% ethanol extracts of *C. orchioides* and *C. latifolia* plant parts.

Table S1. The chemical compounds identified in all sample extracts of *Curculigo orchioides* and *Curculigo latifolia*.

RT [min]	Metabolite name	Molecul Formula	$[M + H]^+$ $[M - H]^-$	Obs. Mass	Cal. Mass	Error mass (ppm)	MS/MS Fragments	Chemical Type
				**ROBM (Rhizome Orchioides Barru)**				
0.80	Unknown-1*	$C_{10}H_{13}N_4O_9$	$[M + H]^+$	333.068	333.068	0.00	-	-
0.88	Crassifoside I	$C_{23}H_{24}O_{11}$	$[M - H]^-$	475.128	475.132	-8.11	457.114; 445.114; 387.108	Phenolic
0.88	Ethyl 3,4-dihydroxybenzoate	$C_9H_{10}O_4$	$[M - H]^-$	181.059	181.058	5.49	153.019; 151.003; 137.024; 135.008	Phenolic
0.88	1,1-Bis(3,4-dihydroxyphenyl)-1-(2-furan)-methane	$C_{17}H_{14}O_5$	$[M - H]^-$	297.082	297.084	-7.81	281.045; 269.081	Phenolic
1.20	Unknown-2	$C_4H_{12}N_6$	$[M+2H]^{2+}$	144.113	144.112	6.94	112.087; 70.066	-
1.37	Unknown-3	$C_{30}H_{26}O$	$[M + H]^+$	402.198	402.198	0.00	-	-
2.85	Unknown-4	CH_2NO_7	$[M + H]^+$	139.982	139.983	-7.14	-	-
3.12	Unknown-5	$C_{12}H_{22}O_{16}$	$[M - H]^-$	422.092	422.091	2.37	-	-
10.15	Orchioside B	$C_{23}H_{26}O_{10}$	$[M + H]^+$	463.149	463.153	-6.89	350.143; 213.143	Phenolic glycosides
12.31	Curculigoside B	$C_{21}H_{24}O_{11}$	$[M + H]^+$	453.136	453.132	8.26	290.271; 276.244	Phenolic glycosides
13.17	Unknown-6	$C_9H_{11}O_5$	$[M + H]^+$	199.059	199.060	-5.02	181.049; 128.951	-
13.48	Unknown-7	$C_{13}H_{28}O_{20}$	$[M + H]^+$	504.122	504.117	0.01	-	-
13.49	Unknown-8	$C_{36}H_{31}O_{10}$	$[M - H]^-$	623.195	623.192	0.01	-	-
15.11	(1S,2R)-O-Methylnyasicoside	$C_{24}H_{28}O_{11}$	$[M + H]^+$	493.167	493.163	7.60	475.159; 313.117	Norlignan glycosides
17.83	Curculigosaponin G	$C_{42}H_{70}O_{13}$	$[M + H]^+$	783.489	783.482	9.04	621.867; 459.726	Cycloartane
18.99	Lycorine	$C_{16}H_{17}NO_4$	$[M - H]^-$	286.118	286.116	7.86	270.076; 268.097; 258.076; 256.097	Alkaloid
20.26	Unknown-9	$C_{65}H_{56}O_3$	$[M + H]^+$	884.428	884.423	5.65	-	-
20.67	Curculigosaponin C	$C_{41}H_{68}O_{13}$	$[M + H]^+$	769.470	769.466	5.50	606.413; 474.370	Cycloartane
24.66	Orchioside B	$C_{23}H_{26}O_{10}$	$[M - H]^-$	461.157	461.153	9.35	443.134; 433.150; 371.113	Phenolic glycosides
26.03	Unknown-10	$C_7H_{12}NO_{12}$	$[M - H]^-$	302.038	302.036	6.62	-	-
30.90	Unknown-11	$C_{12}H_4O_5$	$[M + H]^+$	228.007	228.005	8.77	-	-
				**ROMB (Rhizome Orchioides Maros)**				
0.90	Crassifoside I	$C_{23}H_{24}O_{11}$	$[M - H]^-$	475.1285	475.132	-7.06	457.114; 445.114; 387.108	Phenolic
1.51	Unknown-12	$C_6H_{15}N_2O_{18}$	$[M + H]^+$	403.032	403.032	0.00	-	-
2.76	Orcinol glycoside	$C_{13}H_{18}O_7$	$[M + H]^+$	287.1083	287.105	10.63	269.100; 256.121; 254.993; 251.093; 237.020	Phenolic glycosides
3.06	Lycorine	$C_{16}H_{17}NO_4$	$[M + H]^+$	288.1157	288.116	-0.18	270.113; 252.102; 240.102; 216.102	Alkaloid
3.60	Orchioside A	$C_{22}H_{26}O_{11}$	$[M - H]^-$	465.1489	465.148	3.00	449.108; 447.129; 435.129	Phenolic glycosides
9.22	Unknown-13	$C_{28}H_{19}O_{14}$	$[M + H]^+$	579.079	579.077	3.45	-	-
9.83	Orchioside B	$C_{23}H_{26}O_{10}$	$[M + H]^+$	463.149	463.153	-8.40	350.143; 213.143	Phenolic glycosides
12.89	Unknown-14	$C_{45}H_{11}O_2$	$[M + H]^+$	583.077	583.076	1.72	-	-
15.08	(1S,2R)-O-Methylnyasicoside	$C_{24}H_{28}O_{11}$	$[M + H]^+$	493.167	493.163	8.00	475.159; 313.117	Norlignan glycosides
15.88	Unknown-15	$C_2H_{10}N_5O_5$	$[M + H]^+$	184.068	184.068	0.00	-	-
16.49	Curculigosaponin C	$C_{41}H_{68}O_{13}$	$[M + H]^+$	769.474	769.466	10.70	606.413; 474.370	Cycloartane
20.32	Curculigosaponin G	$C_{42}H_{70}O_{13}$	$[M + H]^+$	783.480	783.482	-2.32	621.867; 459.726	Cycloartane

Table S1. *Cont.*

20.32	Curculigosaponin H	$C_{47}H_{78}O_{17}$	$[M + H]^+$	915.529	915.524	5.93	751.350; 589.137; 458.650	Cycloartane
21.02	Unknown-16	$C_{17}H_{15}O_3$	$[M + H]^+$	267.102	267.102	0.00	-	-
21.86	Curculigoside B	$C_{21}H_{24}O_{11}$	$[M + H]^+$	453.134	453.132	4.07	290.271; 276.244	Phenolic glycosides
24.58	Orchioside B	$C_{23}H_{26}O_{10}$	$[M - H]^-$	461.157	461.153	10.21	443.134; 433.150; 371.113	Phenolic glycosides
25.48	Curculigosaponin C	$C_{41}H_{68}O_{13}$	$[M - H]^-$	767.465	767.466	-1.91	693.421; 659.416	Cycloartane
26.79	Unknown-17	$C_{43}H_{82}O_{17}$	$[M + H]^+$	870.561	870.555	6.89	-	-
31.11	2,4-Dichloro-5-methoxy-3-methylphenol	$C_8H_8Cl_2O_2$	$[M - H]^-$	204.992	204.990	9.60	176.987; 174.972; 154.990; 150.972	Phenolic
**ROGM (Rhizome Orchioides Gowa)**								
0.79	Unknown-18	$C_{11}H_{20}N_{11}O_5$	$[M + H]^+$	386.165	386.164	2.59	-	-
0.90	1,1-Bis(3,4-dihydroxyphenyl)-1-(2-furan)-methane	$C_{17}H_{14}O_5$	$[M - H]^-$	297.082	297.084	-8.82	281.045; 269.081	Phenolic
3.12	Orcinol glycoside	$C_{13}H_{18}O_7$	$[M + H]^+$	287.107	287.105	7.15	269.100; 256.121; 254.993; 251.093; 237.020	Phenolic glycosides
3.12	Lycorine	$C_{16}H_{17}NO_4$	$[M + H]^+$	288.116	288.116	0.86	270.113; 252.102; 240.102; 216.102	Alkaloid
3.19	Curculigoside B	$C_{21}H_{24}O_{11}$	$[M + H]^+$	453.134	453.132	5.39	290.271; 276.244	Phenolic glycosides
3.65	Orchioside A	$C_{22}H_{26}O_{11}$	$[M - H]^-$	465.150	465.148	4.29	449.108; 447.129; 435.129	Phenolic glycosides
3.65	Curculigoside C	$C_{22}H_{26}O_{12}$	$[M - H]^-$	481.139	481.142	-8.15	463.124; 453.140; 451.124	Phenolic glycosides
11.14	Unknown-19	$C_{29}H_{29}O_4$	$[M + H]^+$	441.207	441.206	2.27	-	-
15.13	(1S,2R)-O-Methylnyasicoside	$C_{24}H_{28}O_{11}$	$[M + H]^+$	493.168	493.163	10.23	475.159; 313.117	Norlignan glycosides
15.80	Curculigosaponin G	$C_{42}H_{70}O_{13}$	$[M + H]^+$	783.486	783.482	5.47	621.867; 459.726	Cycloartane
15.96	Unknown-20	$C_{37}H_{16}O_2$	$[M + H]^+$	492.116	492.115	2.03	-	-
18.20	Curculigosaponin I	$C_{48}H_{80}O_{18}$	$[M + H]^+$	945.540	945.534	5.46	783.009; 621.867; 458.726	Cycloartane
18.21	Curculigosaponin I	$C_{48}H_{80}O_{18}$	$[M - H]^-$	943.527	943.534	-8.20	783.009; 621.867; 458.726	Cycloartane
22.39	Unknown-21	$C_{47}H_{47}O_2$	$[M + H]^+$	643.358	643.358	0.00	-	-
23.90	Unknown-22	$C_{44}H_{56}O_3$	$[M + H]^+$	632.426	632.423	4.74	-	-
24.03	Unknown-23	$C_{17}H_{46}N_{13}O_4$	$[M + H]^+$	496.379	496.380	-2.01	-	-
24.27	Unknown-24	$C_{16}H_{33}O_{10}$	$[M + H]^+$	385.210	385.207	7.79	-	-
31.19	2,4-Dichloro-5-methoxy-3-methylphenol	$C_8H_8Cl_2O_2$	$[M - H]^-$	204.992	204.990	10.57	176.987; 174.972; 154.990; 150.972	Phenolic
**RLSP (Rhizome Latifolia Palangka)**								
0.73	Unknown-25***	$H_7N_5O_4$	$[M + H]^+$	141.050	141.049	7.09	-	-
0.83	Unknown-26**	$C_{16}H_{11}O_2$	$[M + H]^+$	235.078	235.076	8.51	-	-
1.32	Curculigoside B	$C_{21}H_{24}O_{11}$	$[M + H]^+$	453.136	453.132	8.48	290.271; 276.244	Phenolic glycosides
1.40	Crassifoside I	$C_{23}H_{24}O_{11}$	$[M - H]^-$	475.127	475.132	-9.59	457.114; 445.114; 387.108	Phenolic
1.47	2,4-Dichloro-5-methoxy-3-methylphenol	$C_8H_8Cl_2O_2$	$[M - H]^-$	204.990	204.990	-2.60	176.987; 174.972; 154.990; 150.972	Phenolic
3.13	Lycorine	$C_{16}H_{17}NO_4$	$[M + H]^+$	288.116	288.116	-0.88	270.113; 252.102; 240.102; 216.102	Alkaloid
3.14	Unknown-27	$C_{11}H_{16}N_9O_2$	$[M + H]^+$	306.143	306.142	3.27	-	-
3.15	Unknown-28	$C_{23}H_{39}O$	$[M - H]^-$	331.301	331.300	3.02	-	-
3.71	Orchioside A	$C_{22}H_{26}O_{11}$	$[M - H]^-$	465.150	465.148	6.01	449.108; 447.129; 435.129	Phenolic glycosides
4.13	Unknown-29	$C_{14}H_{14}N_{11}O_2$	$[M - H]^-$	368.133	368.133	0.00	-	-

Table S1. *Cont.*

5.01	Unknown-30	$C_{23}H_{10}O_{12}$	$[M-H]^-$	478.019	478.017	4.18	-	-
9.44	Unknown-31	$C_{12}H_{20}O_{19}$	$[M+H]^+$	468.058	468.059	-2.14	-	-
10.13	Orchioside B	$C_{23}H_{26}O_{10}$	$[M+H]^+$	463.150	463.153	-6.24	350.143; 213.143	Phenolic glycosides
10.88	Unknown-32	$C_{21}H_{27}O_{10}$	$[M+H]^+$	439.158	439.160	-4.55	-	-
10.90	Curculigoside B	$C_{21}H_{24}O_{11}$	$[M-H]^-$	451.136	451.132	8.08	433.114; 423.129	Phenolic glycosides
11.82	Unknown-33	$C_{27}H_{27}O_{16}$	$[M-H]^-$	607.132	607.130	3.29	-	-
12.72	Unknown-34	$C_{69}H_{41}O_5$	$[M+H]^+$	949.296	949.295	1.05	-	-
15.47	(1S,2R)-O-Methylnyasicoside	$C_{24}H_{28}O_{11}$	$[M+H]^+$	493.167	493.163	8.00	475.159; 313.117	Norlignan glycosides
17.82	Unknown-35	$C_{24}H_{26}O_{19}$	$[M-H]^-$	618.106	618.107	-1.62	-	-
18.16	Unknown-36	$C_{25}H_{29}O$	$[M+H]^+$	345.224	345.221	8.69	-	-
19.02	Lycorine	$C_{16}H_{17}NO_4$	$[M-H]^-$	286.118	286.116	8.90	270.076; 268.097; 258.076; 256.097	Alkaloid
20.25	Falcarinol	$C_{17}H_{24}O$	$[M-H]^-$	244.182	244.183	-4.10	227.144; 215.144; 201.128; 187.149	Organic hydroxy
24.50	Orchioside B	$C_{23}H_{26}O_{10}$	$[M-H]^-$	461.157	461.153	9.13	443.134; 433.150; 371.113	Phenolic glycosides
24.87	Curculigosaponin G	$C_{42}H_{70}O_{13}$	$[M+H]^+$	783.483	783.482	2.15	621.867; 459.726	Cycloartane
26.09	Unknown-37	$C_{26}H_{45}O_5$	$[M-H]^-$	437.325	437.327	-4.57	-	-
26.84	Unknown-38	$C_{51}H_{84}O_{14}$	$[M+H]^+$	920.585	920.586	-1.09	-	-
29.03	3-O-B-D-Glucopyranosyl sitosterol	$C_{35}H_{60}O_6$	$[M-H]^-$	575.434	575.439	-7.93	559.400; 547.400; 477.322	Sitosterol
31.17	2,4-Dichloro-5-methoxy-3-methylphenol	$C_8H_8Cl_2O_2$	$[M-H]^-$	204.992	204.990	10.57	176.987; 174.972; 154.990; 150.972	Phenolic
**RLSB (Rhizome Latifolia Biji Nangka)**								
0.80	Unknown-39*	$C_{10}H_{13}N_4O_9$	$[M+H]^+$	333.068	333.068	0.00	-	-
0.88	Crassifoside I	$C_{23}H_{24}O_{11}$	$[M-H]^-$	475.129	475.132	-5.80	457.114; 445.114; 387.108	Phenolic
0.91	1,1-Bis(3,4-dihydroxyphenyl)-1-(2-furan)-methane	$C_{17}H_{14}O_5$	$[M-H]^-$	297.081	297.084	-9.83	281.045; 269.081	Phenolic
0.96	Lycorine	$C_{16}H_{17}NO_4$	$[M+H]^+$	288.116	288.116	0.86	270.113; 252.102; 240.102; 216.102	Alkaloid
1.44	2,4-Dichloro-5-methoxy-3-methylphenol	$C_8H_8Cl_2O_2$	$[M-H]^-$	204.990	204.990	-1.14	176.987; 174.972; 154.990; 150.972	Phenolic
2.59	Curculigoside B	$C_{21}H_{24}O_{11}$	$[M-H]^-$	451.135	451.132	6.75	433.114; 423.129	Phenolic glycosides
2.98	Unknown-40	$C_{32}H_{31}O_{19}$	$[M-H]^-$	719.146	719.146	0.00	-	-
3.20	Lycorine	$C_{16}H_{17}NO_4$	$[M+H]^+$	288.115	288.116	-2.61	270.113; 252.102; 240.102; 216.102	Alkaloid
3.78	Orchioside A	$C_{22}H_{26}O_{11}$	$[M-H]^-$	465.149	465.148	3.00	449.108; 447.129; 435.129	Phenolic glycosides
5.12	Unknown-41	$C_{52}H_{51}O_{20}$	$[M-H]^-$	995.294	995.297	-3.01	-	-
5.96	Unknown-42	$C_{37}H_{33}O_{11}$	$[M+H]^+$	653.206	653.202	6.12	-	-
6.47	Curculigoside B	$C_{21}H_{24}O_{11}$	$[M+H]^+$	453.132	453.132	0.10	290.271; 276.244	Phenolic glycosides
6.94	Unknown-43	$C_{43}H_{22}O_{14}$	$[M+H]^+$	762.105	762.100	6.56	-	-
8.04	Longirostrerone A	$C_{32}H_{40}O_7$	$[M+H]^+$	536.273	536.277	-7.46	519.274; 501.263; 475.284; 447.180	-
10.36	Unknown-44	$C_{62}H_{52}O_{11}$	$[M+H]^+$	972.345	972.350	-5.14	-	-
12.55	Unknown-45	$C_{66}H_{40}O_{10}$	$[M-H]^-$	992.265	992.262	3.02	-	-
12.71	Unknown-46	$C_{24}H_{41}O_{14}$	$[M+H]^+$	553.247	553.249	-3.62	-	-
14.20	Unknown-47	$C_{29}H_{37}O_{16}$	$[M+H]^+$	641.207	641.208	-1.56	-	-

Table S1. *Cont.*

15.60	Sarmentosumin D	$C_{50}H_{42}O_9$	$[M + H]^+$	786.279	786.283	-5.09	769.279; 759.295; 691.232	-
16.51	Tenuifoliside B	$C_{30}H_{36}O_{17}$	$[M – H]^-$	668.197	668.195	2.99	385.114; 369.082; 367.103; 355.103	-
16.88	Unknown-48	$C_{20}H_{23}O_5$	$[M + H]^+$	343.154	343.155	-2.91	-	-
17.42	Unknown-49	$C_{29}H_{37}O_{12}$	$[M – H]^-$	577.229	577.228	1.73	-	-
18.04	Unknown-50	$C_{40}H_{37}O_{17}$	$[M + H]^+$	789.199	789.203	-5.07	-	-
19.03	Lycorine	$C_{16}H_{17}NO_4$	$[M – H]^-$	286.118	286.116	6.11	270.076; 268.097; 258.076; 256.097	Alkaloid
19.64	Unknown-51	$C_{58}H_{37}O_{13}$	$[M – H]^-$	941.225	941.223	2.12	-	-
19.66	Unknown-52	$C_{33}H_{17}O_2$	$[M + H]^+$	445.123	445.122	2.25	-	-
19.72	Curculigoside C	$C_{22}H_{26}O_{12}$	$[M + H]^+$	483.144	483.142	3.69	465.139; 421.112	Phenolic glycosides
21.00	Unknown-53	$C_{28}H_{47}O_6$	$[M + H]^+$	479.341	479.337	8.34	-	-
23.25	Curculigosaponin G	$C_{42}H_{70}O_{13}$	$[M – H]^-$	781.476	781.482	-7.06	621.867; 459.726	Cycloartane
23.51	Ecdysterone	$C_{27}H_{44}O_7$	$[M + H]^+$	480.308	480.309	-2.08	473.333; 467.106; 454.833; 451.750	
24.62	Curculigoside B	$C_{21}H_{24}O_{11}$	$[M – H]^-$	451.128	451.132	-9.21	433.114; 423.129	Phenolic glycosides
24.62	Orchioside B	$C_{23}H_{26}O_{10}$	$[M – H]^-$	461.157	461.153	10.00	443.134; 433.150; 371.113	Phenolic glycosides
26.11	Unknown-54	$C_{11}H_5O_{18}$	$[M – H]^-$	424.949	424.948	2.35	-	-
28.64	Unknown-55	$C_{52}H_{102}O_{11}$	$[M + H]^+$	902.733	902.742	-9.97	-	-
29.29	Unknown-56	$C_{41}H_{73}O_6$	$[M + H]^+$	661.537	661.540	-4.53	-	-
**RLSK (Rhizome Latifolia Puncak)**								
0.73	Unknown-57***	$H_7N_5O_4$	$[M + H]^+$	141.050	141.049	7.09	-	-
0.80	Unknown-58*	$C_{10}H_{13}N_4O_9$	$[M + H]^+$	333.068	333.068	0.00	-	-
0.91	1,1-Bis(3,4-dihydroxyphenyl)-1-(2-furan)-methane	$C_{17}H_{14}O_5$	$[M – H]^-$	297.082	297.084	-8.14	281.045; 269.081	Phenolic
0.93	Lycorine	$C_{16}H_{17}NO_4$	$[M + H]^+$	288.116	288.116	1.21	270.113; 252.102; 240.102; 216.102	Alkaloid
1.45	2,4-Dichloro-5-methoxy-3-methylphenol	$C_8H_8Cl_2O_2$	$[M – H]^-$	204.989	204.990	-3.57	176.987; 174.972; 154.990; 150.972	Phenolic
2.70	Unknown-59	$C_{24}H_{27}O_{14}$	$[M – H]^-$	539.140	539.140	0.00	-	-
2.78	Lycorine	$C_{16}H_{17}NO_4$	$[M + H]^+$	288.115	288.116	-2.61	270.113; 252.102; 240.102; 216.102	Alkaloid
2.90	Unknown-60	$C_{48}H_{28}O$	$[M – H]^-$	620.214	620.214	0.00	-	-
3.12	Orcinol glycoside	$C_{13}H_{18}O_7$	$[M + H]^+$	287.107	287.105	7.50	269.100; 256.121; 254.993; 251.093; 237.020	Phenolic glycosides
3.15	Unknown-61	$C_{65}H_{107}O_2$	$[M + H]^+$	919.827	919.827	0.00	-	-
3.17	Unknown-62	$C_{64}H_{101}O_3$	$[M – H]^-$	917.777	917.775	2.18	-	-
6.38	Curculigoside B	$C_{21}H_{24}O_{11}$	$[M + H]^+$	453.132	453.132	-0.12	290.271; 276.244	Phenolic glycosides
7.77	Unknown-63	$C_{17}H_{36}O_{14}$	$[M + H]^+$	464.213	464.210	6.46	-	-
12.54	Unknown-64	$C_{36}H_{19}O_{14}$	$[M – H]^-$	675.082	675.077	7.41	-	-
14.14	Orchioside A	$C_{22}H_{26}O_{11}$	$[M + H]^+$	467.153	467.148	10.91	305.144; 291.144	Phenolic glycosides
14.48	Unknown-65	$C_{22}H_{27}O_{10}$	$[M + H]^+$	451.158	451.160	-4.43	-	-
15.52	Unknown-66	$C_{47}H_{21}O_{19}$	$[M – H]^-$	889.065	889.068	-3.37	-	-
23.38	Unknown-67	$C_{36}H_{43}O_9$	$[M – H]^-$	619.289	619.292	-4.84	-	-
24.68	Curculigoside B	$C_{21}H_{24}O_{11}$	$[M – H]^-$	451.128	451.132	-9.21	433.114; 423.129	Phenolic glycosides

Table S1. *Cont.*

24.68	Orchioside B	$C_{23}H_{26}O_{10}$	$[M - H]^-$	461.157	461.153	10.00	443.134; 433.150; 371.113	Phenolic glycosides
24.77	Unknown-68	$C_{61}H_{82}O_5$	$[M + H]^+$	894.618	894.616	2.24	-	-
25.21	Unknown-69	$C_{34}H_{62}N_{15}O_9$	$[M - H]^-$	824.485	824.485	0.00	-	-
26.12	Unknown-70	$C_{48}H_{79}O_{12}$	$[M - H]^-$	847.558	847.557	1.18	-	-
26.50	Unknown-71	$C_{55}H_{75}O_3$	$[M + H]^+$	783.572	783.571	1.28	-	-
27.87	Unknown-72	$C_{45}H_{55}O_6$	$[M + H]^+$	691.399	691.399	0.00	-	-
30.89	Curculigosaponin C	$C_{41}H_{68}O_{13}$	$[M - H]^-$	767.464	767.466	-3.22	693.421; 659.416	Cycloartane
30.92	Unknown-73	$C_{42}H_{77}O_{20}$	$[M - H]^-$	901.501	901.501	0.00	-	-
				**LOBM (Leaf Orchioides Barru)**				
0.80	Unknown-74*	$C_{10}H_{13}N_4O_9$	$[M + H]^+$	333.068	333.068	0.00	-	-
1.52	Unknown-75	$CH_{18}N_{20}O_7$	$[M + H]^+$	422.167	422.166	2.37	-	-
4.06	Unknown-76	$C_{13}H_9O_{11}$	$[M + H]^+$	341.014	341.014	0.00	-	-
8.77	Unknown-77	$C_{13}H_{30}N_5O_{11}$	$[M - H]^-$	432.194	432.194	0.00	-	-
13.31	Unknown-78	$C_{17}H_{32}N_7O_{19}$	$[M + H]^+$	638.175	638.175	0.00	-	-
14.27	Orcinol glycoside	$C_{13}H_{18}O_7$	$[M + H]^+$	287.103	287.105	-9.22	269.100; 256.121; 254.993; 251.093; 237.020	Phenolic glycosides
14.67	Unknown-79	$C_7H_5N_{13}O_4$	$[M+2H]^{2+}$	335.059	335.058	2.98	-	-
14.85	Unknown-80	$C_{42}H_{12}O_2$	$[M + H]^+$	548.083	548.084	-1.82	-	-
15.90	Unknown-81	$C_{59}H_{46}O_{15}$	$[M - H]^-$	994.279	994.284	-5.03	-	-
19.80	Curculigosaponin G	$C_{42}H_{70}O_{13}$	$[M + H]^+$	783.486	783.482	5.21	621.867; 459.726	Cycloartane
15.91	Unknown-82	$C_{66}H_{35}O_{11}$	$[M + H]^+$	987.222	987.223	-1.01	-	-
20.05	Curculigosaponin I	$C_{48}H_{80}O_{18}$	$[M + H]^+$	945.537	945.534	3.03	783.009; 621.867; 458.726	Cycloartane
21.09	Unknown-83	$C_{26}H_{58}N_{17}O_7$	$[M + H]^+$	720.471	720.470	1.39	-	-
22.20	Unknown-84	$C_{30}H_{61}O_{19}$	$[M - H]^-$	725.379	725.381	-2.76	-	-
23.27	Cayaponoside B3	$C_{34}H_{50}O_{11}$	$[M - H]^-$	634.333	634.335	-3.15	471.275; 455.243; 415.212; 401.196	-
23.90	Unknown-85	$C_{42}H_{51}O_6$	$[M + H]^+$	651.365	651.368	-4.61	-	-
24.85	Unknown-86	$C_{55}H_{85}O_{12}$	$[M - H]^-$	937.604	937.604	0.00	-	-
25.04	Unknown-87	$C_{57}H_{86}N_5O_5$	$[M + H]^+$	920.663	920.662	1.09	-	-
27.69	Curculigosaponin C	$C_{41}H_{68}O_{13}$	$[M + H]^+$	769.467	769.466	1.34	606.413; 474.370	Cycloartane
29.01	Curculigosaponin H	$C_{47}H_{78}O_{17}$	$[M + H]^+$	915.526	915.524	1.78	751.350; 589.137; 458.650	Cycloartane
30.90	Unknown-88	$C_{54}H_{68}O_2$	$[M - H]^-$	748.515	748.522	-9.35	-	-
				**LOMB (Leaf Orchioides Maros)**				
0.83	Unknown-89**	$C_{16}H_{11}O_2$	$[M + H]^+$	235.078	235.076	8.51	-	-
0.90	1,1-Bis(3,4-dihydroxyphenyl)-1-(2-furan)-methane	$C_{17}H_{14}O_5$	$[M - H]^-$	297.081	297.084	-9.15	281.045; 269.081	Phenolic
1.19	Unknown-90	$C_{18}H_{37}N_8O_{12}$	$[M + H]^+$	557.253	557.253	0.00	-	-
1.46	2,4-Dichloro-5-methoxy-3-methylphenol	$C_8H_8Cl_2O_2$	$[M - H]^-$	204.990	204.990	-2.60	176.987; 174.972; 154.990; 150.972	Phenolic
1.58	Laciniatoside V	$C_{27}H_{38}O_{14}$	$[M + H]^+$	586.227	586.226	1.71	569.222; 407.170; 393.154; 343.175	-
2.06	Salidroside	$C_{14}H_{20}O_7$	$[M + H]^+$	300.123	300.121	6.66	290.946; 288.967; 283.031	-

Table S1. *Cont.*

2.64	Emodin Dianthrone	$C_{30}H_{20}O_8$	$[M-H]^-$	508.117	508.116	1.97	491.077; 467.077; 441.097; 425.066	-
3.02	Unknown-91	$C_{55}H_{19}O_8$	$[M-H]^-$	807.105	807.108	-3.72	-	-
5.26	Unknown-92	$C_2H_2N_5O_{12}$	$[M+H]^+$	287.967	287.969	-6.95	-	-
5.93	Unknown-93	$C_{34}H_{13}N_{20}O_2$	$[M+H]^+$	733.153	733.153	0.00	-	-
5.95	Unknown-94	$C_{36}H_{24}O_{19}$	$[M-H]^-$	760.092	760.091	1.32	-	-
6.72	Eucommicin A	$C_{32}H_{36}O_{18}$	$[M-H]^-$	708.185	708.190	-7.06	689.172; 645.182; 619.203; 617.187	-
7.54	Unknown-95	$C_5H_{15}N_5O_{12}$	$[M+H]^+$	337.070	337.072	-5.93	-	-
9.38	Unknown-96	$C_{33}H_{33}O_7$	$[M+H]^+$	541.224	541.223	1.85	-	-
10.75	Unknown-97	$C_{33}H_{53}O_{15}$	$[M-H]^-$	689.336	689.338	-2.90	-	-
13.21	Unknown-98	$C_{42}H_{20}O_6$	$[M+H]^+$	620.129	620.126	4.84	-	-
13.24	Orchioside B	$C_{23}H_{26}O_{10}$	$[M+H]^+$	463.149	463.153	-8.61	350.143; 213.143	Phenolic glycosides
13.32	Unknown-99	$C_{37}H_{39}N_{14}O_{20}$	$[M-H]^-$	999.247	999.246	1.00	-	-
13.68	Unknown-100	$C_{20}H_{31}O_{16}$	$[M-H]^-$	527.164	527.161	5.69	-	-
14.87	Unknown-101	$C_{53}H_{61}O_{18}$	$[M-H]^-$	985.387	985.386	1.01	-	-
15.45	(1S,2R)-O-Methylnyasicoside	$C_{24}H_{28}O_{11}$	$[M+H]^+$	493.168	493.163	8.81	475.159; 313.117	Norlignan glycosides
15.66	Unknown-102	$C_{66}H_{46}O_9$	$[M+H]^+$	982.316	982.314	2.04	-	-
15.91	Unknown-103	$C_{66}H_{42}O_{10}$	$[M-H]^-$	994.278	994.278	0.00	-	-
15.93	Unknown-104	$C_{55}H_{39}O_{18}$	$[M+H]^+$	987.219	987.214	5.06	-	-
16.74	Theanaphthoquinone	$C_{28}H_{22}O_{11}$	$[M+H]^+$	534.115	534.116	-1.87	517.112; 507.128; 477.118	Quinones
17.43	Unknown-105	$C_{38}H_{35}N_{18}O_{16}$	$[M-H]^-$	999.248	999.248	0.00	-	-
17.84	Unknown-106	$C_{51}H_{68}O_9$	$[M+H]^+$	824.485	824.486	-1.21	-	-
18.98	Unknown-107	$C_{61}H_{37}O_{14}$	$[M-H]^-$	993.215	993.218	-3.02	-	-
19.32	Curculigosaponin C	$C_{41}H_{68}O_{13}$	$[M+H]^+$	769.471	769.466	6.67	606.413; 474.370	Cycloartane
19.53	Curculigosaponin I	$C_{48}H_{80}O_{18}$	$[M+H]^+$	945.537	945.534	2.60	783.009; 621.867; 458.726	Cycloartane
19.65	Unknown-108	$C_{59}H_{41}O_{15}$	$[M-H]^-$	989.242	989.245	-3.03	-	-
19.66	Unknown-109	$C_{48}H_{55}O_{20}$	$[M+H]^+$	951.328	951.329	-1.05	-	-
19.99	Unknown-110	$C_{56}H_{78}O_{15}$	$[M-H]^-$	990.529	990.534	-5.05	-	-
20.09	Lycorine	$C_{16}H_{17}NO_4$	$[M-H]^-$	286.115	286.116	-1.93	270.076; 268.097; 258.076; 256.097	Alkaloid
20.32	Curculigosaponin G	$C_{42}H_{70}O_{13}$	$[M+H]^+$	783.481	783.482	-1.17	621.867; 459.726	Cycloartane
20.55	Curculigosaponin I	$C_{48}H_{80}O_{18}$	$[M-H]^-$	943.527	943.534	-7.67	783.009; 621.867; 458.726	Cycloartane
21.23	Unknown-111	$C_{30}H_{41}O_{11}$	$[M+H]^+$	577.268	577.265	5.20	-	-
22.08	Unknown-112	$C_{34}H_{43}O_{13}$	$[M+H]^+$	659.269	659.270	-1.52	-	-
22.88	Unknown-113	$C_{38}H_{37}N_3O_3$	$[M+H]^+$	583.283	583.283	0.00	-	-
23.99	Unknown-114	$C_{44}H_{42}N_{20}O_3$	$[M+H]^+$	898.375	898.375	0.00	-	-
24.00	Unknown-115	$C_{59}H_{77}O_{10}$	$[M-H]^-$	945.549	945.552	-3.17	-	-
24.16	Unknown-116	$C_{52}H_{73}O_{11}$	$[M+H]^+$	873.516	873.515	1.14	-	-
24.59	Unknown-117	$C_{52}H_{77}O_{13}$	$[M-H]^-$	909.535	909.536	-1.10	-	-

Table S1. *Cont.*

24.63	Orchioside B	$C_{23}H_{26}O_{10}$	$[M-H]^-$	461.157	461.153	10.21	443.134; 433.150; 371.113	Phenolic glycosides
25.22	Unknown-118	$C_{32}H_{64}N_9O_{10}$	$[M-H]^-$	734.477	734.478	-1.36	-	-
25.72	Unknown-119	$C_{50}H_{89}O_{19}$	$[M-H]^-$	993.599	993.599	0.00	-	-
26.49	Adonixanthin diglucoside	$C_{52}H_{74}O_{13}$	$[M+H]^+$	906.513	906.513	0.00	889509; 729.472; 727.456; 627.368	Glucosides
26.82	Unknown-120	$C_{57}H_{71}O_5$	$[M+H]^+$	835.532	835.530	2.39	-	-
27.70	Unknown-121	$C_{54}H_{73}O_{12}$	$[M+H]^+$	913.511	913.510	1.09	-	-
29.05	Curculigosaponin H	$C_{47}H_{78}O_{17}$	$[M+H]^+$	915.524	915.524	-0.19	751.350; 589.137; 458.650	Cycloartane
29.28	Unknown-122	$C_{45}H_{79}O_{14}$	$[M-H]^-$	843.548	843.546	2.37	-	-
29.37	3-O-B-D-Glucopyranosyl sitosterol	$C_{35}H_{60}O_6$	$[M-H]^-$	575.433	575.439	-10.89	559.400; 547.400; 477.322	Sitosterol
30.32	Unknown-123	$C_{56}H_{94}O_{14}$	$[M+H]^+$	990.663	990.664	-1.01	-	-
30.94	Unknown-124	$C_{60}H_{69}O_{11}$	$[M-H]^-$	965.487	965.484	3.11	-	-
				**LOGM (Leaf Orchioides Gowa)**				
0.83	Unknown-125**	$C_{16}H_{11}O_2$	$[M+H]^+$	235.078	235.076	8.51	-	-
0.87	1,1-Bis(3,4-dihydroxyphenyl)-1-(2-furan)-methane	$C_{17}H_{14}O_5$	$[M-H]^-$	297.082	297.084	-8.48	281.045; 269.081	Phenolic
0.91	Crassifoside I	$C_{23}H_{24}O_{11}$	$[M-H]^-$	475.130	475.132	-4.33	457.114; 445.114; 387.108	Phenolic
1.82	Unknown-126	$CH_{11}N_{13}O$	$[M+H]^+$	221.122	221.121	4.52	-	-
1.41	Orchioside B	$C_{23}H_{26}O_{10}$	$[M+H]^+$	463.155	463.153	4.56	350.143; 213.143	Phenolic glycosides
1.46	2,4-Dichloro-5-methoxy-3-methylphenol	$C_8H_8Cl_2O_2$	$[M-H]^-$	204.989	204.990	-3.57	176.987; 174.972; 154.990; 150.972	Phenolic
3.13	Unknown-127	$C_{17}H_{17}O_{15}$	$[M-H]^-$	461.059	461.057	4.34	-	-
8.88	Crassifoside I	$C_{23}H_{24}O_{11}$	$[M-H]^-$	475.127	475.132	-9.80	457.114; 445.114; 387.108	Phenolic
13.52	Khainaoside C	$C_{25}H_{28}O_{11}$	$[M+H]^+$	504.157	504.162	-9.92	487.159; 309.096; 265.070; 181.085	Glucosides
14.92	Pelargonidin 3-O-(6-O-Malonyl-Beta-D-Glucoside)	$C_{24}H_{23}O_{13}$	$[M-H]^-$	519.110	519.114	-7.71	285.170; 257.175; 243.159	Flavonoid
15.48	(1S,2R)-O-Methylnyasicoside	$C_{24}H_{28}O_{11}$	$[M+H]^+$	493.166	493.163	6.38	475.159; 313.117	Norlignan glycosides
15.84	Unknown-128	$C_{33}H_{19}O_{19}$	$[M+H]^+$	559.104	559.104	0.00	-	-
16.76	Unknown-129	$C_{31}H_{11}O_{11}$	$[M+H]^+$	559.031	559.032	-1.79	-	-
17.46	Asiatic acid	$C_{30}H_{48}O_5$	$[M+H]^+$	488.350	488.351	-2.05	473.326; 463.341; 459.310; 433.294	-
19.04	Unknown-130	$C_{21}H_{34}O_{18}$	$[M+H]^+$	576.177	576.178	-1.74	-	-
19.66	Unknown-131	$C_2H_{15}N_{10}O_8$	$[M+H]^+$	307.107	307.107	0.00	-	-
19.67	Unknown-132	$C_{25}H_{25}O_9$	$[M-H]^-$	469.149	469.149	0.00	-	-
19.67	Orchioside A	$C_{22}H_{26}O_{11}$	$[M+H]^+$	467.153	467.148	10.91	305.144; 291.144	Phenolic glycosides

Table S1. *Cont.*

19.79	Curculigosaponin G	$C_{42}H_{70}O_{13}$	$[M + H]^+$	783.487	783.482	6.87	621.867; 459.726	Cycloartane
20.04	Curculigosaponin I	$C_{48}H_{80}O_{18}$	$[M + H]^+$	945.534	945.534	-0.67	783.009; 621.867; 458.726	Cycloartane
20.30	Unknown-133	$C_{43}H_{61}O_{19}$	$[M - H]^-$	881.381	881.381	0.00	-	-
23.20	Unknown-134	$C_{42}H_{69}O_{13}$	$[M - H]^-$	781.473	781.474	-1.28	-	-
24.51	Orchioside B	$C_{23}H_{26}O_{10}$	$[M - H]^-$	461.157	461.153	8.70	443.134; 433.150; 371.113	Phenolic glycosides
25.03	Unknown-135	$C_{30}H_{49}O_{5}$	$[M + H]^+$	527.898	527.899	-1.89	-	-
25.26	Curculigosaponin G	$C_{42}H_{70}O_{13}$	$[M - H]^-$	781.483	781.482	2.03	621.867; 459.726	Cycloartane
25.57	Unknown-136	$C_{49}H_{77}O_{13}$	$[M - H]^-$	873.535	873.536	-1.14	-	-
26.83	Unknown-137	$C_{28}H_{54}N_{15}O$	$[M + H]^+$	616.463	616.463	0.00	-	-
27.98	Curculigosaponin H	$C_{47}H_{78}O_{17}$	$[M + H]^+$	915.526	915.524	1.78	751.350; 589.137; 458.650	Cycloartane
30.39	Unknown-138	$C_{64}H_{92}O_{3}$	$[M + H]^+$	908.700	908.701	-1.10	-	-
31.34	2,4-Dichloro-5-methoxy-3-methylphenol	$C_{8}H_{8}Cl_{2}O_{2}$	$[M - H]^-$	204.992	204.990	8.62	176.987; 174.972; 154.990; 150.972	Phenolic
**LLSP (Leaf Latifolia Palangka)**								
0.83	Unknown-139**	$C_{16}H_{11}O_{2}$	$[M + H]^+$	235.078	235.076	8.51	-	-
1.27	Unknown-140	$C_{20}H_{29}O_{14}$	$[M - H]^-$	493.154	493.156	-4.06	-	-
1.47	2,4-Dichloro-5-methoxy-3-methylphenol	$C_{8}H_{8}Cl_{2}O_{2}$	$[M - H]^-$	204.990	204.990	-3.09	176.987; 174.972; 154.990; 150.972	Phenolic
2.82	Unknown-141	$C_{45}H_{23}O_{17}$	$[M - H]^-$	835.098	835.094	4.79	-	-
3.05	Curculigoside B	$C_{21}H_{24}O_{11}$	$[M + H]^+$	453.127	453.132	-10.05	290.271; 276.244	Phenolic glycosides
3.07	Unknown-142	$C_{57}H35O_{11}$	$[M - H]^-$	895.217	895.218	-1.12	-	-
4.90	Unknown-143	$C_{44}H_{27}O_{15}$	$[M - H]^-$	795.135	795.135	0.00	-	-
6.87	Unknown-144	$C_{47}H_{53}O_{16}$	$[M - H]^-$	873.336	873.333	3.44	-	-
6.89	Unknown-145	$C_{69}H_{30}O_{3}$	$[M + H]^+$	906.218	906.219	-1.10	-	-
7.14	Orchioside A	$C_{22}H_{26}O_{11}$	$[M - H]^-$	465.143	465.148	-9.25	449.108; 447.129; 435.129	Phenolic glycosides
8.24	Unknown-146	$C_{10}H_{15}N_{2}O_{20}$	$[M - H]^-$	483.022	483.022	0.00	-	-
9.27	Unknown-147	$C_{51}H_{26}O_{9}$	$[M - H]^-$	782.159	782.158	1.28	-	-
10.04	Unknown-148	$C_{28}H_{39}O_{14}$	$[M - H]^-$	599.233	599.234	-1.67	-	-
10.06	Unknown-149	$C_{69}H_{39}O_{3}$	$[M + H]^+$	915.290	915.289	1.09	-	-
10.28	Unknown-150	$C_{29}H_{48}N_{8}O_{20}$	$[M - H]^-$	828.299	828.298	1.21	-	-
10.91	Curculigoside B	$C_{21}H_{24}O_{11}$	$[M - H]^-$	451.136	451.132	10.07	433.114; 423.129	Phenolic glycosides
13.31	Unknown-151	$C_{31}H_{35}O_{16}$	$[M + H]^+$	663.191	663.193	-3.02	-	-
14.57	Unknown-152	$C_{41}H_{31}O_{12}$	$[M - H]^-$	715.179	715.182	-4.19	-	-

Table S1. *Cont.*

14.59	Unknown-153	$C_{42}H_{46}N_{12}O_{17}$	$[M + H]^+$	990.310	990.310	0.00	-	-
17.37	Unknown-154	$C_{43}H_{53}O_{20}$	$[M - H]^-$	889.311	889.313	-2.25	-	-
17.98	Unknown-155	$C_{58}H_{60}O_5$	$[M + H]^+$	836.445	836.444	1.20	-	-
18.87	Unknown-156	$C_{47}H_{79}O_{19}$	$[M + H]^+$	947.522	947.521	1.06	-	-
19.81	Unknown-157	$C_{55}H_{72}O15$	$[M - H]^-$	972.485	972.487	-2.06	-	-
20.22	Unknown-158	$C_{34}H_{37}O_{11}$	$[M + H]^+$	621.232	621.234	-3.22	-	-
20.43	Curculigosaponin G	$C_{42}H_{70}O_{13}$	$[M + H]^+$	783.487	783.482	7.00	621.867; 459.726	Cycloartane
21.11	Curculigoside C	$C_{22}H_{26}O_{12}$	$[M - H]^-$	481.141	481.142	-2.12	463.124; 453.140; 451.124	Phenolic glycosides
22.16	Unknown-159	$C_{16}H_{34}N_7O_9$	$[M - H]^-$	468.242	468.242	0.00	-	-
22.17	Unknown-160	$C_{39}H_{59}O_{15}$	$[M + H]^+$	767.387	767.385	2.61	-	-
22.89	Unknown-161	$C_{68}H_{62}O_7$	$[M + H]^+$	990.452	990.449	3.03	-	-
23.26	Unknown-162	$C_{58}H_{47}O_{12}$	$[M + H]^+$	935.310	935.307	3.21	-	-
23.34	Unknown-163	$C_{25}H_{66}N_{16}O_{19}$	$[M - H]^-$	894.469	894.468	1.12	-	-
24.36	Curculigosaponin I	$C_{48}H_{80}O_{18}$	$[M - H]^-$	943.530	943.534	-5.02	783.009; 621.867; 458.726	Cycloartane
24.48	Unknown-164	$C_{48}H_{61}N_5O_{15}$	$[M - H]^-$	947.416	947.416	0.00	-	-
24.52	Orchioside B	$C_{23}H_{26}O_{10}$	$[M - H]^-$	461.157	461.153	9.35	443.134; 433.150; 371.113	Phenolic glycosides
24.84	Nyasicoside	$C_{23}H_{26}O_{11}$	$[M - H]^-$	477.150	477.148	6.07	459.129; 449.145; 447.129	Phenolic
25.05	Curculigosaponin G	$C_{42}H_{70}O_{13}$	$[M - H]^-$	781.475	781.482	-9.11	621.867; 459.726	Cycloartane
25.33	Unknown-165	$C_{67}H_{76}O_6$	$[M + H]^+$	976.564	976.564	0.00	-	-
25.92	Curculigosaponin H	$C_{47}H_{78}O_{17}$	$[M - H]^-$	913.525	913.524	0.90	751.350; 589.137; 458.650	Cycloartane
26.00	Unknown-166	$C_{58}H_{77}O_{10}$	$[M + H]^+$	933.550	933.552	-2.14	-	-
26.12	Unknown-167	$C_{51}H_{76}N_6O_{10}$	$[M - H]^-$	932.562	932.561	1.07	-	-
26.71	Unknown-168	$C_{49}H_{79}O_9$	$[M + H]^+$	811.575	811.572	3.70	-	-
27.50	1,1-Bis(3,4-dihydroxyphenyl)-1-(2-furan)-methane	$C_{17}H_{14}O_5$	$[M - H]^-$	297.082	297.084	-6.12	281.045; 269.081	Phenolic
27.73	Unknown-169	$C_{59}H_{87}O_{10}$	$[M + H]^+$	955.631	955.629	2.09	-	-
28.03	Curculigosaponin H	$C_{47}H_{78}O_{17}$	$[M + H]^+$	915.520	915.524	-4.23	751.350; 589.137; 458.650	Cycloartane
29.94	3-O-B-D-Glucopyranosyl sitosterol	$C_{35}H_{60}O_6$	$[M - H]^-$	575.434	575.439	-8.46	559.400; 547.400; 477.322	Sitosterol
30.09	Tragopogonsaponin M	$C_{51}H_{76}O_{16}$	$[M + H]^+$	944.515	944.513	2.12	927.510; 913.494; 783.468; 767.473	Pentacyclic triterpenes
30.90	Unknown-170^	$C_{56}H_{77}O_{13}$	$[M + H]^+$	957.534	957.536	-2.09	-	Phenolic

Table S1. *Cont.*

30.92	Unknown-171	$C_{57}H_{65}O_5$	$[M-H]^-$	829.484	829.483	1.21	-	-
**LLSB (Leaf Latifolia Biji Nangka)**								
3.75	Unknown-172	$C_7H_{16}N_5O_9$	$[M-H]^-$	314.094	314.095	-3.18	-	-
5.49	Curculigoside B	$C_{21}H_{24}O_{11}$	$[M+H]^+$	453.137	453.132	10.25	290.271; 276.244	Phenolic glycosides
6.68	Unknown-173	$C_6H_{10}O_{13}$	$[M+H]^+$	290.013	290.012	3.45	-	-
13.32	Unknown-174	$C_{10}H_{12}N_8O_{10}$	$[M-H]^-$	404.067	404.068	-2.47	-	-
14.93	Unknown-176	$C_{15}H_{13}O_8$	$[M+H]^+$	321.063	321.061	6.23	-	-
15.05	Unknown-175	$C_{26}H_{35}O_{16}$	$[M-H]^-$	603.195	603.192	4.97	-	-
16.85	(1S,2R)-O-Methylnyasicoside	$C_{24}H_{28}O_{11}$	$[M+H]^+$	493.168	493.163	10.43	475.159; 313.117	Norlignan glycosides
17.63	Unknown-177	$C_{26}H_{37}O_{19}$	$[M-H]^-$	653.189	653.193	-6.12	-	-
22.25	Unknown-178	$C_{41}H_{45}O_{12}$	$[M-H]^-$	729.289	729.291	-2.74	-	-
22.41	Unknown-179	$C_{47}H_{49}O$	$[M+H]^+$	629.381	629.378	4.77	-	-
23.22	Unknown-180	$C_{50}H_{83}O_{17}$	$[M-H]^-$	955.561	955.563	-2.09	-	-
24.20	Unknown-181	$C_{40}H_{44}O_9$	$[M+H]^+$	668.294	668.298	-5.99	-	-
24.51	Curculigoside B	$C_{21}H_{24}O_{11}$	$[M-H]^-$	451.128	451.132	-7.66	433.114; 423.129	Phenolic glycosides
24.52	Unknown-182	$C_{16}H_{30}O_{15}$	$[M-H]^-$	462.159	462.158	2.16	-	-
24.56	Orchioside B	$C_{23}H_{26}O_{10}$	$[M-H]^-$	461.157	461.153	10.43	443.134; 433.150; 371.113	Phenolic glycosides
24.63	Unknown-183	$C_{14}H_{23}O_{13}$	$[M-H]^-$	399.113	399.114	-2.51	-	-
25.56	3-O-B-D-Glucopyranosyl sitosterol	$C_{35}H_{60}O_6$	$[M-H]^-$	575.442	575.439	5.10	559.400; 547.400; 477.322	Sitosterol
26.80	Curculigosaponin C	$C_{41}H_{68}O_{13}$	$[M-H]^-$	767.463	767.466	-3.48	693.421; 659.416	Cycloartane
28.44	Unknown-184	$C_{60}H_{78}O_6$	$[M+H]^+$	894.580	894.579	1.12	-	-
30.92	Unknown-185	$C_{47}H_{59}O_7$	$[M+H]^+$	735.427	735.426	1.36	-	-
31.14	2,4-Dichloro-5-methoxy-3-methylphenol	$C_8H_8Cl_2O_2$	$[M-H]^-$	204.992	204.990	8.62	176.987; 174.972; 154.990; 150.972	Phenolic
**LLSK (Leaf Latifolia Puncak)**								
0.86	Pothobanoside C	$C_{26}H_{38}O_{16}$	$[M-H]^-$	606.212	606.216	-6.60	443.155; 427.124; 411.129; 359.098	Glycosides
0.89	1,1-Bis(3,4-dihydroxyphenyl)-1-(2-furan)-methane	$C_{17}H_{14}O_5$	$[M-H]^-$	297.082	297.084	-8.82	281.045; 269.081	Phenolic
3.38	Unknown-186	$C_{13}H_4N_{20}$	$[M+H]^+$	440.093	440.092	2.27	-	-
7.89	Curculigoside B	$C_{21}H_{24}O_{11}$	$[M+H]^+$	453.136	453.132	9.81	290.271; 276.244	Phenolic glycosides
12.87	Unknown-187	$C_{52}H_{100}O_{15}$	$[M-H]^-$	964.701	964.706	-5.18	-	-
13.29	Unknown-188	$C_{25}H_{42}O_{15}$	$[M-H]^-$	582.257	582.252	8.59	-	-
13.70	Unknown-189	$C_{26}H_{29}O_{11}$	$[M+H]^+$	517.169	517.171	-3.87	-	-

Table S1. *Cont.*

14.94	Unknown-190	$C_{38}H_{43}O_{16}$	$[M-H]^-$	755.250	755.255	-6.62	-	-
15.03	Unknown-191	$C_{49}H_{27}O_8$	$[M+H]^+$	743.167	743.170	-4.04	-	-
15.51	Unknown-192	$C_{56}H_{21}O_4$	$[M+H]^+$	757.143	757.144	-1.32	-	-
15.84	Unknown-193	$C_{32}H_{59}O_{11}$	$[M-H]^-$	619.406	619.406	0.00	-	-
16.87	Unknown-194	$C_{26}H_{40}N_7O_{18}$	$[M-H]^-$	738.242	738.243	-1.35	-	-
18.34	Unknown-195	$C_{29}H_{51}O_{14}$	$[M+H]^+$	623.329	623.328	1.60	-	-
19.03	Unknown-196	$C_{32}H_{60}O_{13}$	$[M-H]^-$	652.410	652.403	10.73	-	-
19.53	Unknown-197	$C_{49}H_{67}O_4$	$[M+H]^+$	719.506	719.503	4.17	-	-
19.83	Unknown-198	$C_{44}H_{80}O_{14}$	$[M-H]^-$	832.559	832.555	4.80	-	-
20.31	Unknown-199	$C_{48}H_{83}O_{11}$	$[M-H]^-$	835.591	835.594	-3.59	-	-
20.62	Unknown-200	$C_{36}H_{49}O_7$	$[M-H]^-$	593.347	593.348	-1.69	-	-
21.11	Unknown-201	$C_{42}H_{49}O_{15}$	$[M-H]^-$	793.302	793.307	-6.30	-	-
23.24	Unknown-202	$C_{35}H_{47}O_{12}$	$[M+H]^+$	659.308	659.306	3.03	-	-
24.23	Unknown-203	$C_{55}H_{103}O_{11}$	$[M+H]^+$	939.750	939.749	1.06	-	-
24.55	Unknown-204	$C_{55}H_{63}O_{15}$	$[M-H]^-$	963.418	963.417	1.04	-	-
24.56	Orchioside B	$C_{23}H_{26}O_{10}$	$[M-H]^-$	461.157	461.153	8.70	443.134; 433.150; 371.113	Phenolic glycosides
24.59	Curculigoside B	$C_{21}H_{24}O_{11}$	$[M-H]^-$	451.128	451.132	-9.21	433.114; 423.129	Phenolic glycosides
25.17	Unknown-205	$C_{62}H_{64}O_7$	$[M-H]^-$	920.466	920.465	1.09	-	-
25.18	Orchioside A	$C_{22}H_{26}O_{11}$	$[M-H]^-$	465.144	465.148	-8.18	449.108; 447.129; 435.129	Phenolic glycosides
25.66	3-O-B-D-Glucopyranosyl sitosterol	$C_{35}H_{60}O_6$	$[M-H]^-$	575.441	575.439	3.36	559.400; 547.400; 477.322	Sitosterol
25.93	Curculigosaponin H	$C_{47}H_{78}O_{17}$	$[M-H]^-$	913.526	913.524	2.22	751.350; 589.137; 458.650	Cycloartane
26.02	Unknown-206	$C_{40}H_{51}O_{16}$	$[M+H]^+$	787.316	787.317	-1.27	-	-
26.11	Unknown-207	$C_{34}H_{74}N_{11}O_{10}$	$[M-H]^-$	796.562	796.562	0.00	-	-
26.74	Unknown-208	$C_{46}H_{47}O_9$	$[M+H]^+$	743.326	743.322	5.38	-	-
27.04	Unknown-209	$C_{58}H_{107}O_{11}$	$[M-H]^-$	979.782	979.781	1.02	-	-
27.32	Unknown-210	$C_{54}H_{83}O_{12}$	$[M+H]^+$	923.588	923.588	0.00	-	-
30.12	Unknown-211	$C_{63}H_{87}O_5$	$[M+H]^+$	923.655	923.655	0.00	-	-
30.90	Unknown-212^	$C_{56}H_{77}O_{13}$	$[M+H]^+$	957.534	957.536	-2.09	-	-
30.92	Unknown-213	$C_{50}H_{61}O_{13}$	$[M-H]^-$	869.411	869.411	0.00	-	-
31.44	2,4-Dichloro-5-methoxy-3-methylphenol	$C_8H_8Cl_2O_2$	$[M-H]^-$	204.992	204.990	10.57	176.987; 174.972; 154.990; 150.972	Phenolic

Table S1. *Cont.*

**PLSP (Petiole Latifolia Palangka)**								
0.83	Unknown-214**	$C_{16}H_{11}O_2$	$[M + H]^+$	235.078	235.076	8.51	-	-
0.92	1,1-Bis(3,4-dihydroxyphenyl)-1-(2-furan)-methane	$C_{17}H_{14}O_5$	$[M - H]^-$	297.081	297.084	-9.15	281.045; 271.061; 269.081	Phenolic
0.93	Orchioside A	$C_{22}H_{26}O_{11}$	$[M + H]^+$	467.153	467.148	10.69	305.144; 291.144	Phenolic glycosides
0.94	Crassifoside I	$C_{23}H_{24}O_{11}$	$[M - H]^-$	475.129	475.132	-6.85	457.114; 445.114; 387.108	Phenolic
1.48	2,4-Dichloro-5-methoxy-3-methylphenol	$C_8H_8Cl_2O_2$	$[M - H]^-$	204.990	204.990	-1.62	176.987; 174.972; 154.990; 150.972	Phenolic
6.32	Orchioside B	$C_{23}H_{26}O_{10}$	$[M + H]^+$	463.157	463.153	8.87	350.143; 213.143	Phenolic glycosides
9.28	Unknown-215	$C_{18}H_{32}O_{13}$	$[M + H]^+$	456.186	456.184	4.38	-	-
12.55	Breviscapin	$C_{21}H_{18}O_{12}$	$[M - H]^-$	462.078	462.079	-2.16	285.040; 267.029; 257.045; 243.029	Glucuronates
13.29	Unknown-216	$C_{20}H_{11}N_6O_{19}$	$[M - H]^-$	639.007	639.008	-1.56	-	-
21.21	Unknown-217	$C_{31}H_{31}O_{11}$	$[M + H]^+$	579.184	579.186	-3.45	-	-
21.81	Prunin	$C_{21}H_{22}O_{10}$	$[M + H]^+$	434.118	434.121	-6.91	273.075; 153.019; 147.045	-
22.88	Unknown-218	$C_{24}H_{37}O_{13}$	$[M - H]^-$	533.228	533.223	9.38	-	-
23.11	Unknown-219	$C_{40}H_{52}O_7$	$[M + H]^+$	644.376	644.371	7.76	-	-
23.31	Unknown-220	$C_{18}H_{24}O_{16}$	$[M - H]^-$	496.102	496.106	-8.06	-	-
24.53	Curculigoside B	$C_{21}H_{24}O_{11}$	$[M - H]^-$	451.128	451.132	-9.21	433.114; 423.129	Phenolic glycosides
25.49	Curculigosaponin C	$C_{41}H_{68}O_{13}$	$[M - H]^-$	767.467	767.466	0.82	693.421; 659.416	Cycloartane
26.10	Curculigosaponin G	$C_{42}H_{70}O_{13}$	$[M - H]^-$	781.480	781.482	-1.81	621.867; 459.726	Cycloartane
26.70	Unknown-221	$C_{63}H_{74}O_8$	$[M + H]^+$	958.539	958.538	1.04	-	-
28.30	Unknown-222	$C_{62}H_{68}N_7O_3$	$[M + H]^+$	958.538	958.538	0.00	-	-
**PLSB (Petiole Latifolia Biji Nangka)**								
0.88	Vanillin	$C_8H_8O_3$	$[M - H]^-$	151.048	151.047	6.34	150.981; 137.018; 136.015; 129.113	Phenolic
11.54	Orchioside B	$C_{23}H_{26}O_{10}$	$[M - H]^-$	461.148	461.153	-9.30	443.134; 433.150; 371.113	Phenolic glycosides
16.27	Unknown-223	$C_{21}H_{33}O_{15}$	$[M + H]^+$	525.176	525.181	-9.52	-	-
19.22	Neosakuranin	$C_{22}H_{24}O_{10}$	$[M + H]^+$	448.137	448.136	2.23	431.133; 293.138; 287.091; 271.096	-
24.66	Curculigoside B	$C_{21}H_{24}O_{11}$	$[M - H]^-$	451.128	451.132	-9.99	433.114; 423.129	Phenolic glycosides
25.23	Orchioside A	$C_{22}H_{26}O_{11}$	$[M - H]^-$	465.144	465.148	-6.89	449.108; 447.129; 435.129	Phenolic glycosides
25.26	Sophoranone	$C_{30}H_{36}O_4$	$[M - H]^-$	460.262	460.261	2.17	443.222; 391.190; 387.159; 257.190	Flavonoids
25.92	Curculigosaponin H	$C_{47}H_{78}O_{17}$	$[M - H]^-$	913.526	913.524	2.77	751.350; 589.137; 458.650	Cycloartane
30.92	Unknown-224	$C_{11}H_4N_4O_8$	$[M + H]^+$	320.003	320.002	3.12	-	-
31.24	2,4-Dichloro-5-methoxy-3-methylphenol	$C_8H_8Cl_2O_2$	$[M - H]^-$	204.992	204.990	8.62	176.987; 174.972; 154.990; 150.972	Phenolic

Table S1. *Cont.*

**PLSK (Petiole Latifolia Puncak)**								
0.85	Crassifoside I	$C_{23}H_{24}O_{11}$	$[M + H]^+$	475.128	475.131	-8.11	457.114; 447.129; 445.114; 387.108	Phenolic
0.88	1,1-Bis(3,4-dihydroxyphenyl)-1-(2-furan)-methane	$C_{17}H_{14}O_5$	$[M - H]^-$	297.082	297.084	-7.47	281.045; 269.081	Phenolic
1.44	2,4-Dichloro-5-methoxy-3-methylphenol	$C_8H_8Cl_2O_2$	$[M - H]^-$	204.990	204.990	-2.11	176.987; 174.972; 154.990; 150.972	Phenolic
3.14	Lycorine	$C_{16}H_{17}NO_4$	$[M + H]^+$	288.116	288.116	0.17	270.113; 252.102; 240.102; 216.102	Alkaloid
3.68	Orchioside A	$C_{22}H_{26}O_{11}$	$[M - H]^-$	465.150	465.148	5.79	449.108; 447.129; 435.129	Phenolic glycosides
6.09	Unknown-225	$C_{20}H_{25}O_{11}$	$[M + H]^+$	441.136	441.139	-6.80	-	-
6.38	Curculigoside B	$C_{21}H_{24}O_{11}$	$[M + H]^+$	453.133	453.132	2.75	290.271; 276.244	Phenolic glycosides
13.06	Unknown-226	$C_{38}H_{27}N_{18}O_2$	$[M - H]^-$	767.256	767.255	1.30	-	-
19.01	Lycorine	$C_{16}H_{17}NO_4$	$[M - H]^-$	286.118	286.116	8.90	270.076; 268.097; 258.076; 256.097	Alkaloid
23.29	Curculigosaponin G	$C_{42}H_{70}O_{13}$	$[M - H]^-$	781.475	781.482	-8.47	621.867; 459.726	Cycloartane
24.59	Orchioside B	$C_{23}H_{26}O_{10}$	$[M - H]^-$	461.157	461.153	8.70	443.134; 433.150; 371.113	Phenolic glycosides
24.63	Curculigoside B	$C_{21}H_{24}O_{11}$	$[M - H]^-$	451.129	451.132	-5.44	433.114; 423.129	Phenolic glycosides
25.23	Unknown-227	$C_{69}H_{78}O_2$	$[M - H]^-$	938.600	938.600	0.00	-	-
25.53	Unknown-228	$C_{34}H_{41}O_{11}$	$[M + H]^+$	609.272	609.269	4.92	-	-
25.94	Curculigosaponin H	$C_{47}H_{78}O_{17}$	$[M - H]^-$	913.524	913.524	-0.19	751.350; 589.137; 458.650	Cycloartane
29.21	3-O-B-D-Glucopyranosyl sitosterol	$C_{35}H_{60}O_6$	$[M - H]^-$	575.434	575.439	-9.15	559.400; 547.400; 477.322	Sitosterol
30.89	Curculigosaponin C	$C_{41}H_{68}O_{13}$	$[M - H]^-$	767.466	767.466	0.17	693.421; 659.416	Cycloartane
31.03	2,4-Dichloro-5-methoxy-3-methylphenol	$C_8H_8Cl_2O_2$	$[M - H]^-$	204.992	204.990	9.60	176.987; 174.972; 154.990; 150.972	Phenolic

* and ^: same RT and molecule formula

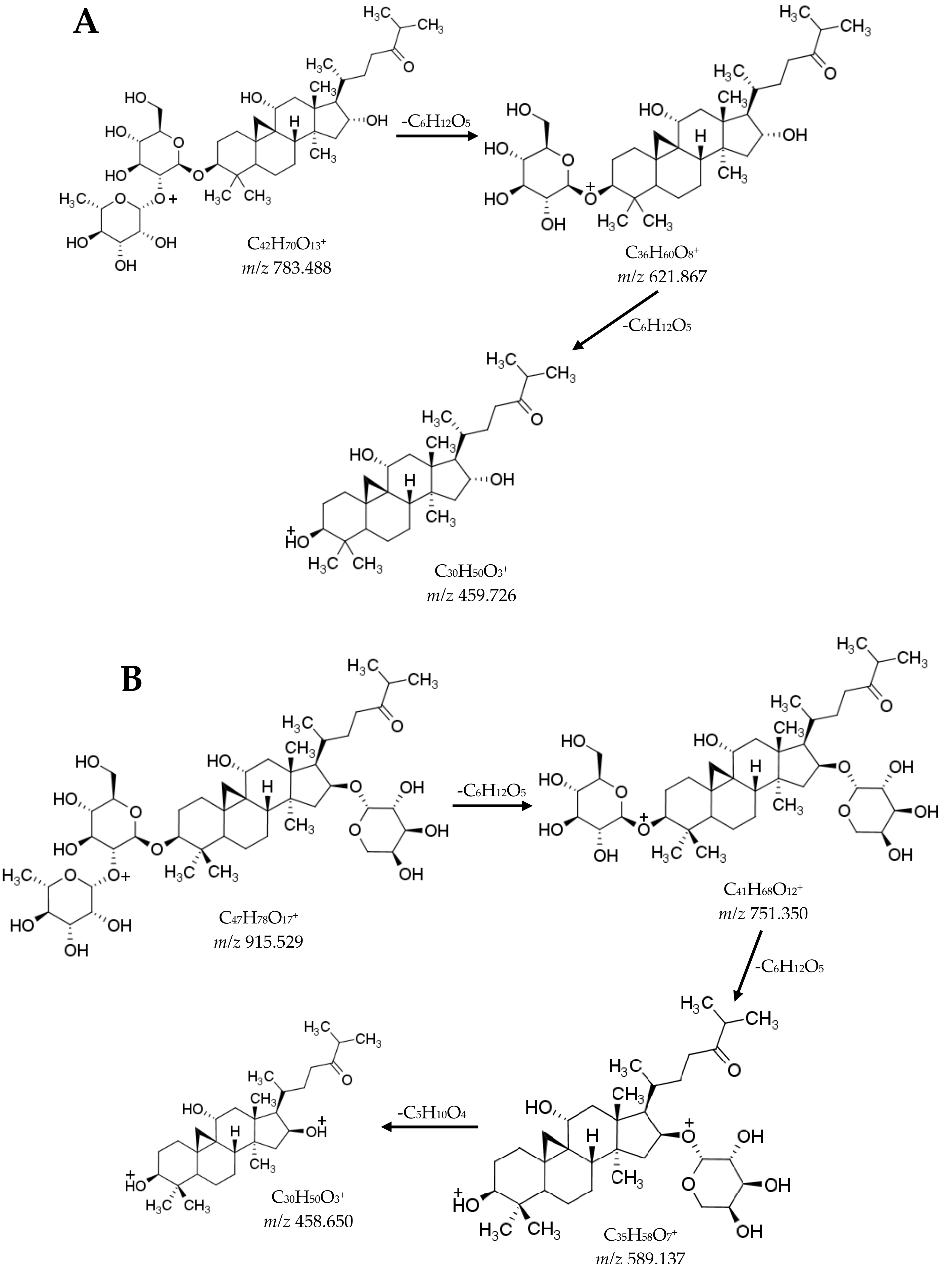
A
-$C_6H_{12}O_5$
$C_{42}H_{70}O_{13}^+$
m/z 783.488
$C_{36}H_{60}O_8^+$
m/z 621.867
-$C_6H_{12}O_5$
$C_{30}H_{50}O_3^+$
m/z 459.726
B
-$C_6H_{12}O_5$
$C_{47}H_{78}O_{17}^+$
m/z 915.529
$C_{41}H_{68}O_{12}^+$
m/z 751.350
-$C_6H_{12}O_5$
-$C_5H_{10}O_4$
$C_{30}H_{50}O_3^+$
m/z 458.650
$C_{35}H_{58}O_7^+$
m/z 589.137

C

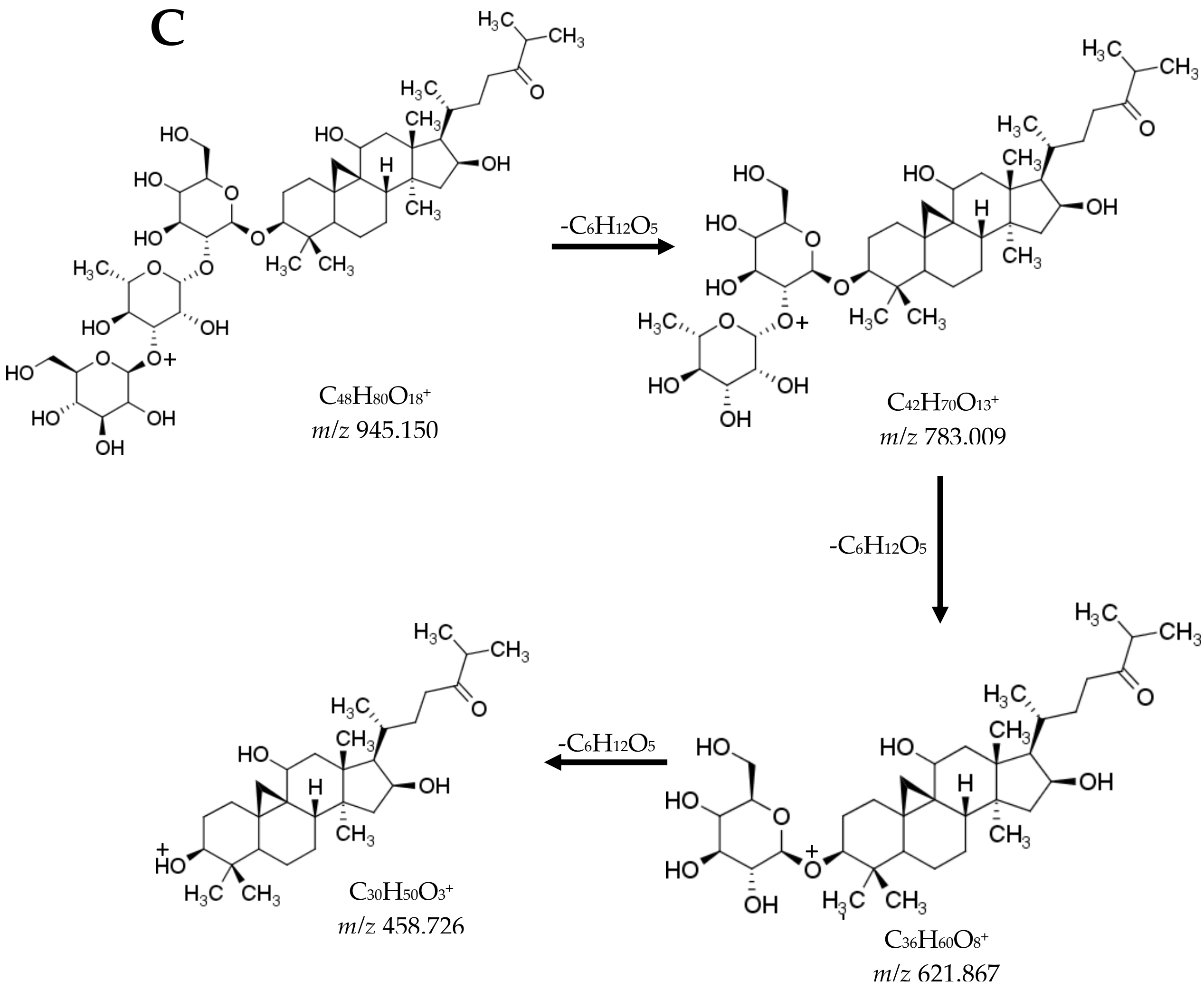

-C6H12O5
C48H80O18+
m/z 945.150
C42H70O13+
m/z 783.009
-C6H12O5
-C6H12O5
C30H50O3+
m/z 458.726
C36H60O8+
m/z 621.867

D

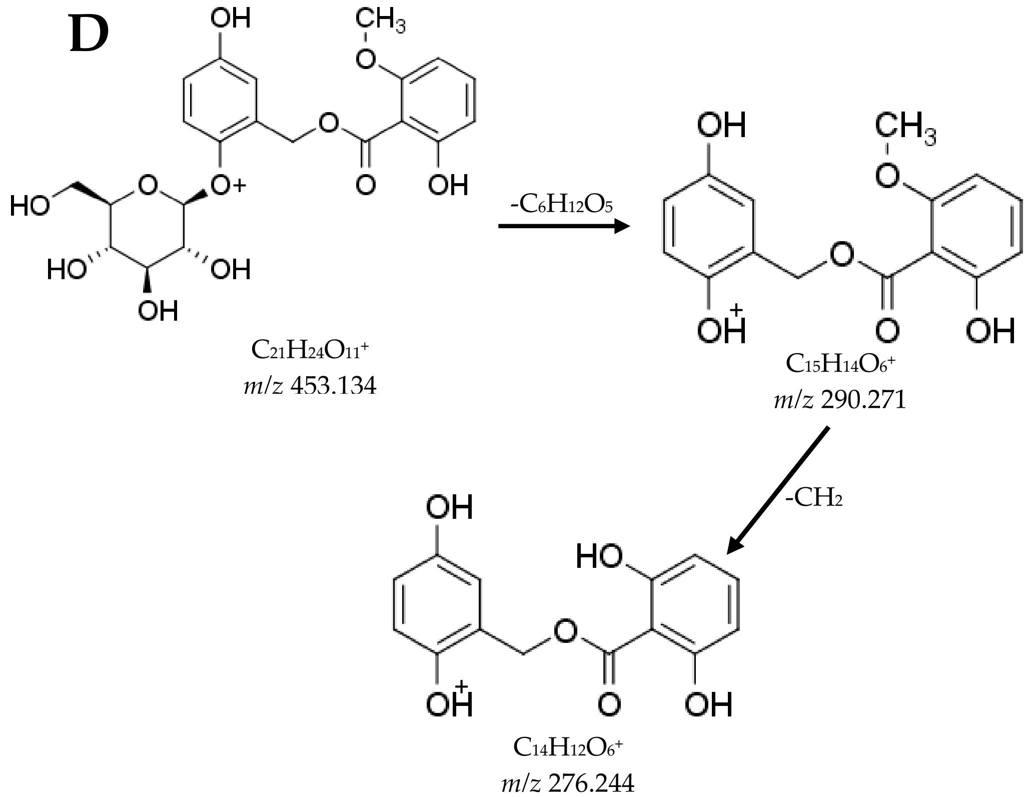

-C6H12O5
C21H24O11+
m/z 453.134
C15H14O6+
m/z 290.271
-CH2
C14H12O6+
m/z 276.244

E

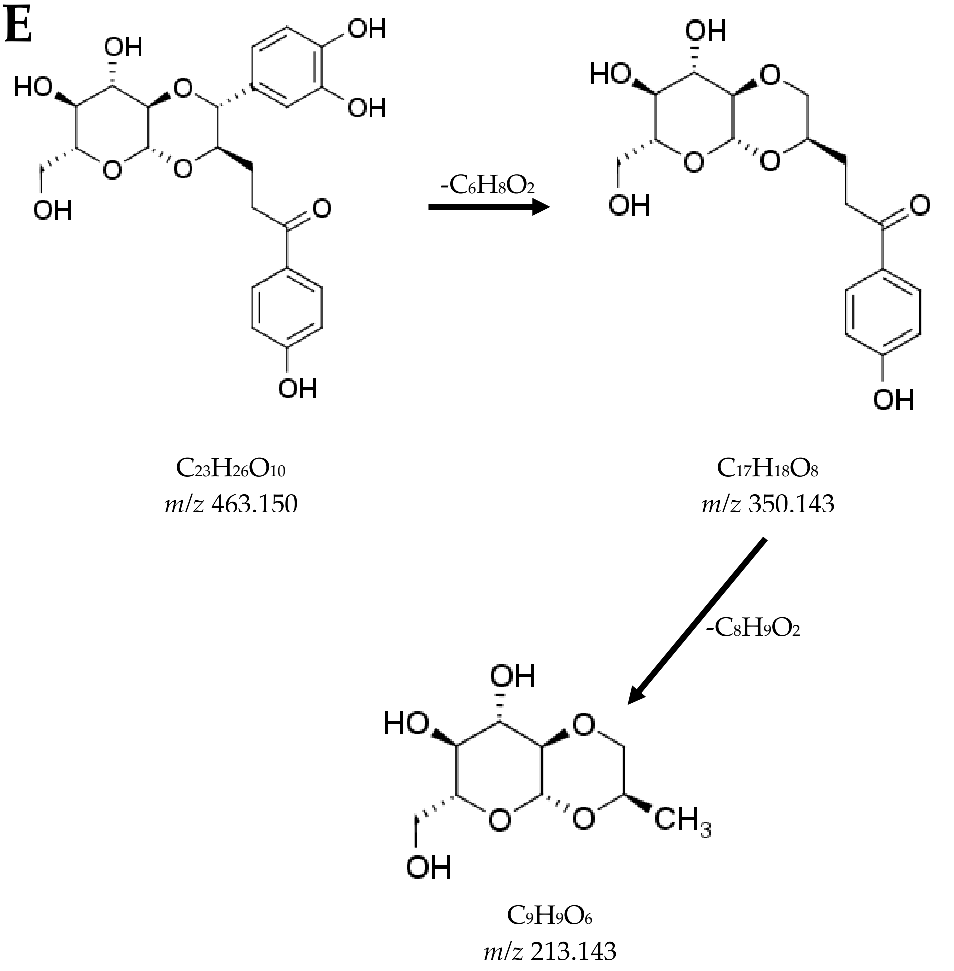

OH
HO
O
OH
OH
O
OH
O
O
OH
-C6H8O2
C23H26O10
m/z 463.150
C17H18O8
m/z 350.143
-C8H9O2
CH3
C9H9O6
m/z 213.143

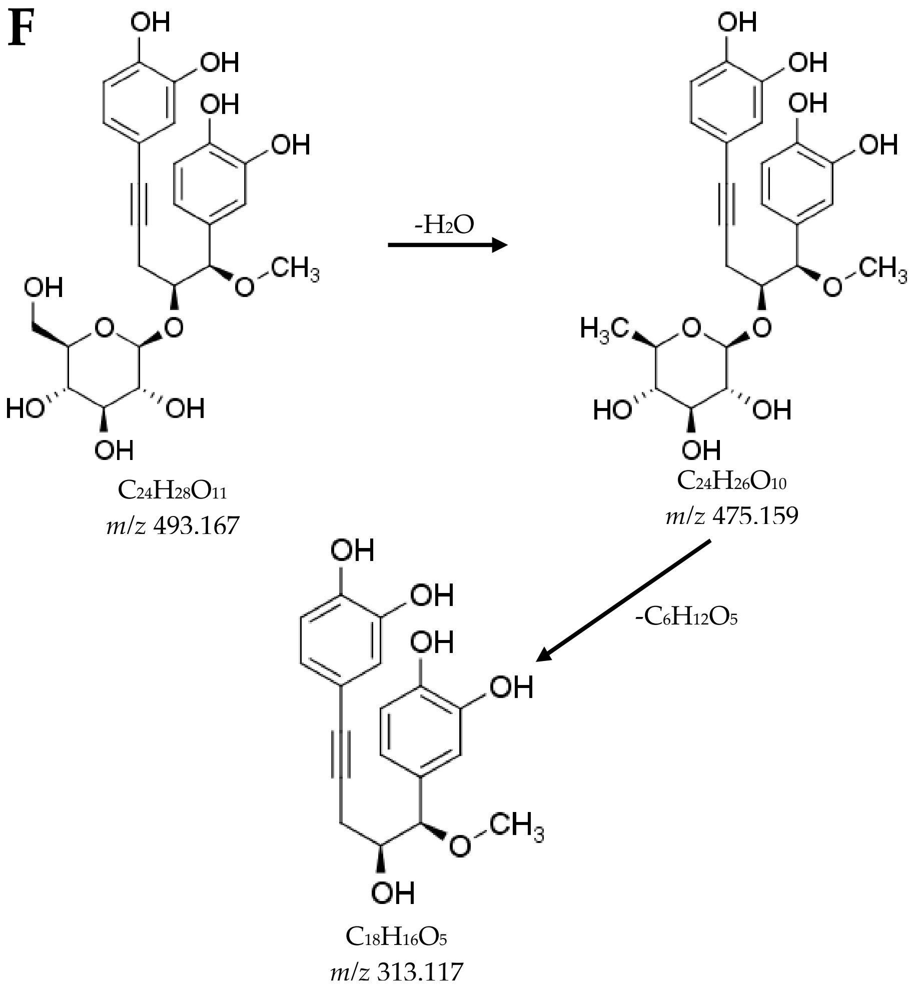

Figure S3. The proposed fragmentation pattern of representative curculigosaponin G (triterpenoid) (**A**), curculigosaponin H (triterpenoid) (**B**), curculigosaponin I (triterpenoid) (**C**), curculigoside B (phenolic) (**D**), orchioiside B (phenolic) (**E**), (1S,2R)-O-Methylnyasicoside (norlignan) (**F**) at ion mode $[M + H]^+$) and $[M - H]^-$) in *C. orchioides* and *C. latifolia*.

A

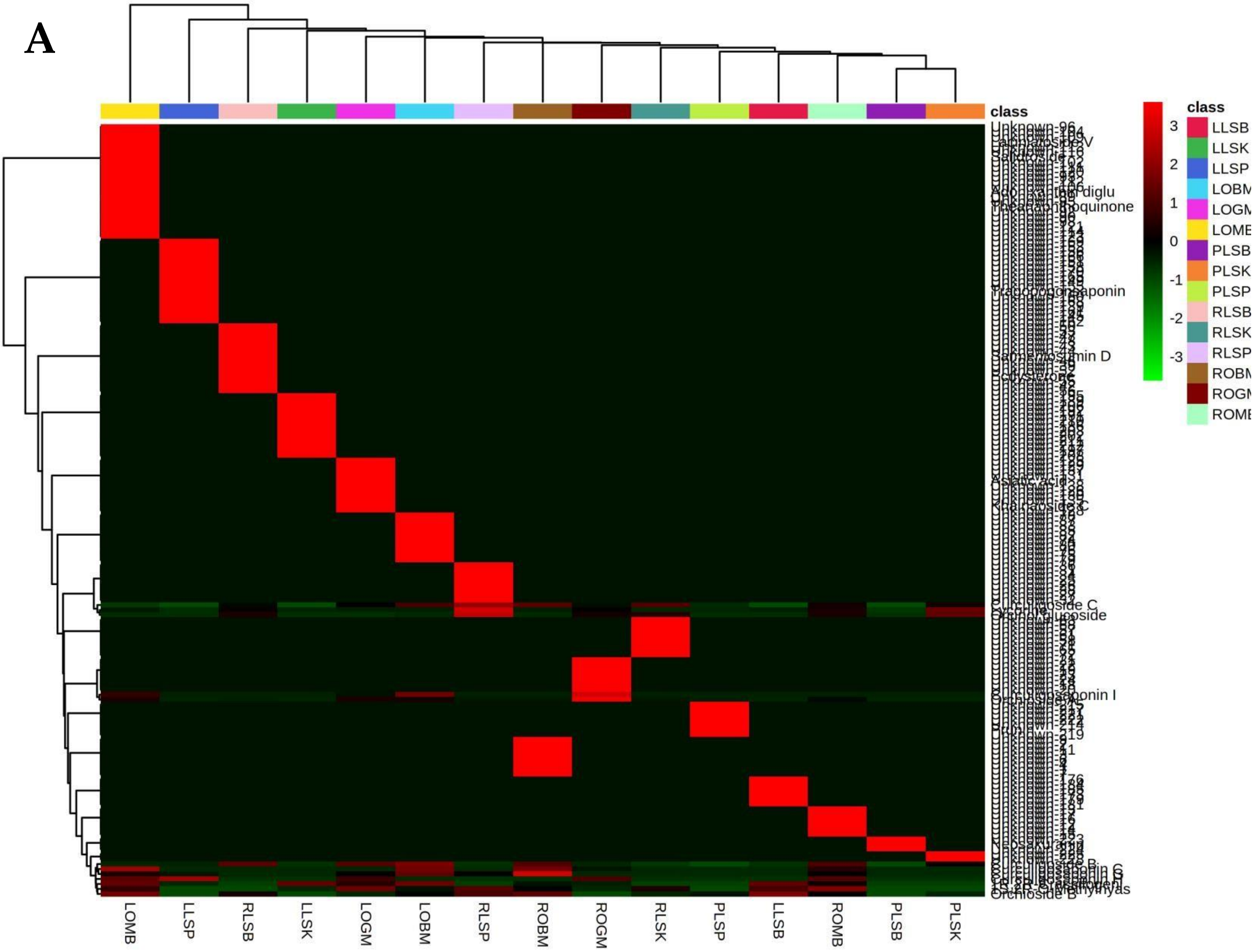
class
LLSB
LLSK
LLSP
LOBM
LOGM
LOME
PLSB
PLSK
PLSP
RLSB
RLSK
RLSP
ROBM
ROGM
ROME
3
2
1
0
-1
-2
-3
LOMB
LLSP
RLSB
LLSK
LOGM
LOBM
RLSP
ROBM
ROGM
RLSK
PLSP
LLSB
ROMB
PLSB
PLSK
Unknown-96
Tracogonsaponin
Orchioside B

B

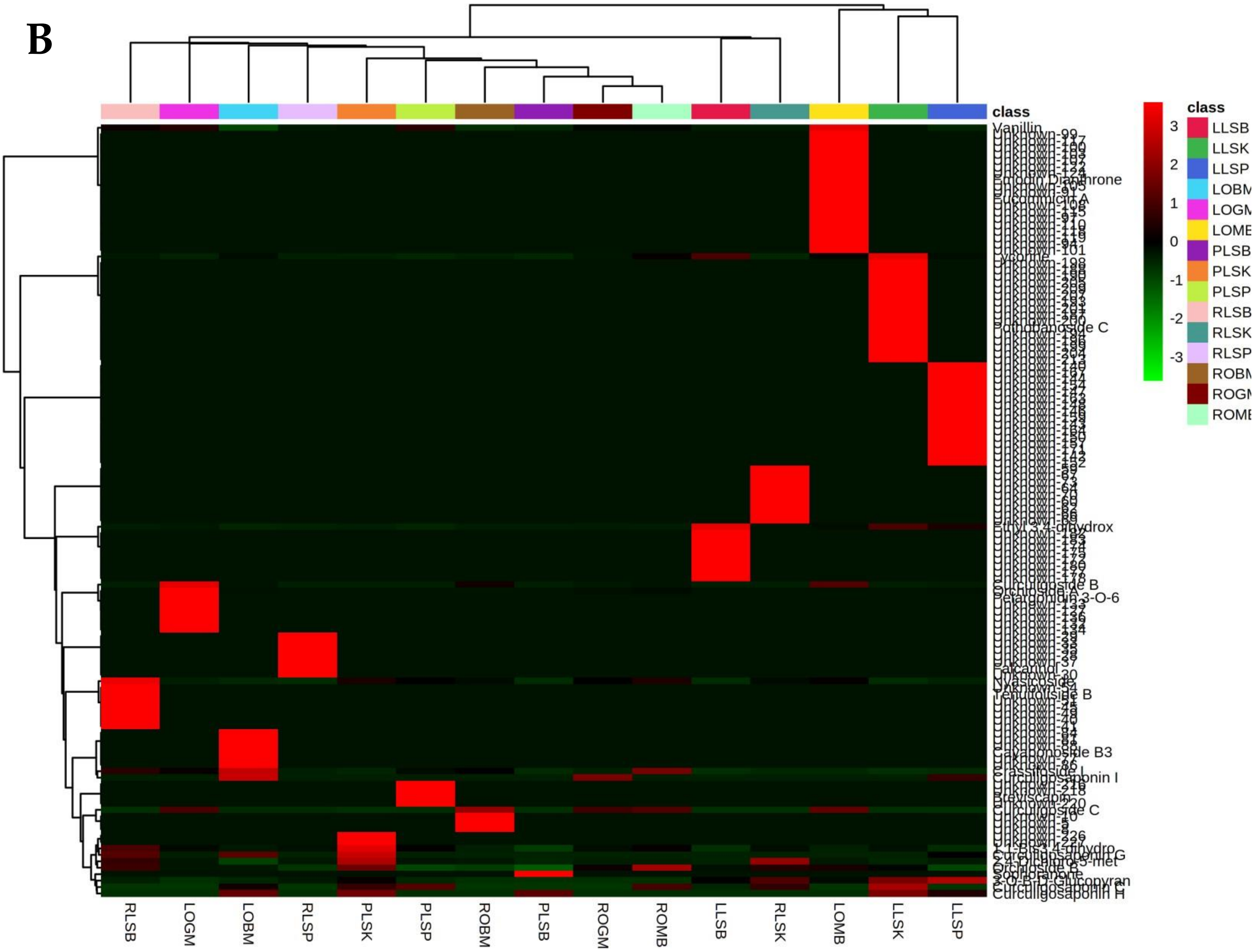

Figure S4. Heatmap correlation between chemical compounds in 70% ethanol extract of *C. orchioides* and *C. latifolia* and their relative abundance. Positive mode (**A**) and negative mode (**B**). The color scale differentiates values: high (red), moderate (black), and low (green).

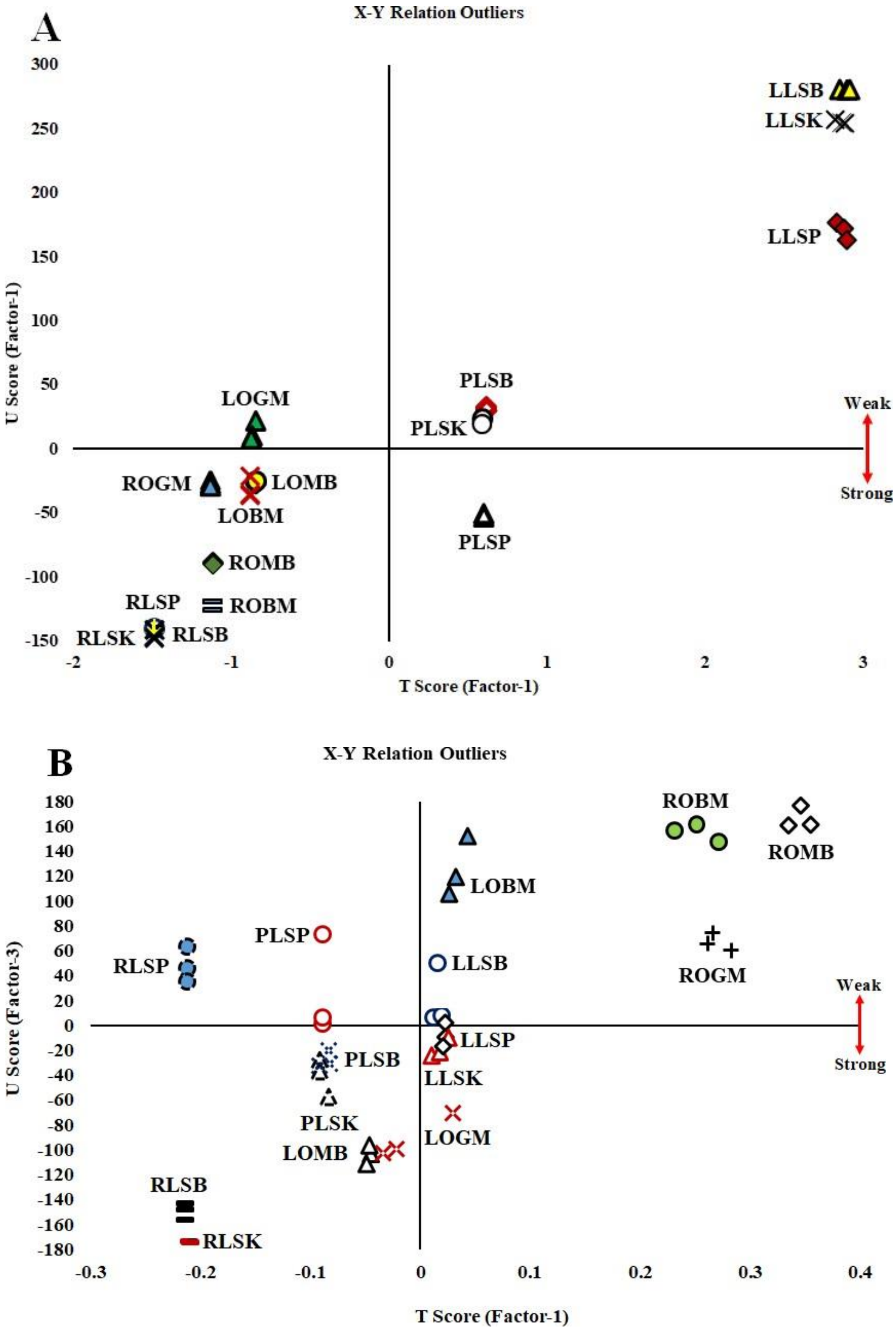

Figure S5. Partial least squares plot x-y relation outliers antioxidant (**A**) and inhibitory of α- glucosidase (**B**) activity of a 70% ethanol extract of *C. orchioides* and *C. latifolia*. Axes and ordinate plots show partial least squares scores for each organ sample.

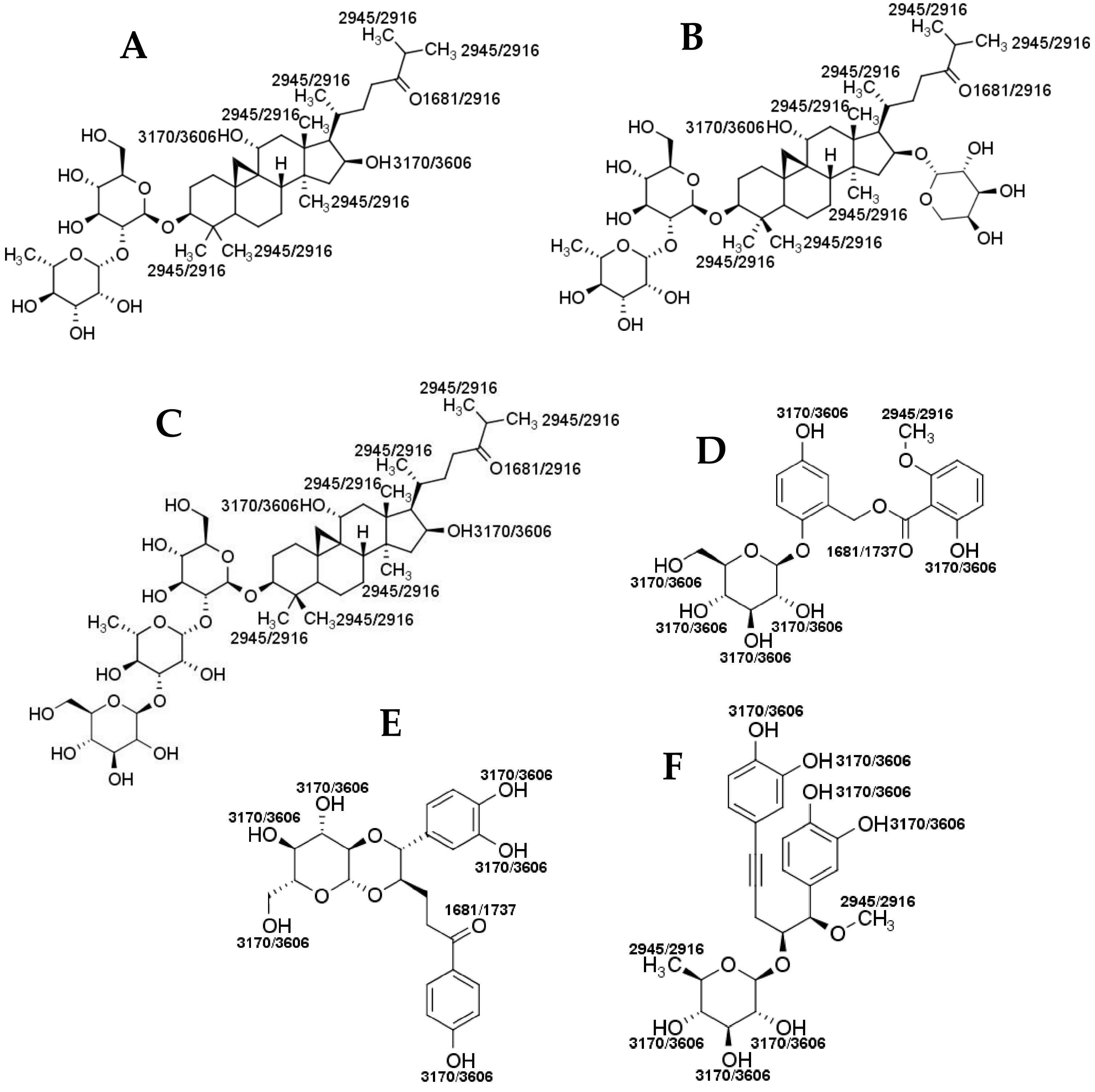

Figure S6. Chemical structure of curculigosaponin G (**A**), curculigosaponin H (**B**), curculigosaponin I (**C**), curculigoside B (**D**), orchioiside B (**E**), and (1S,2R)-O-Methylnyasicoside (**F**) and their functional groups might have a major contribution to the antioxidant activity and the inhibition of α-glucosidase.

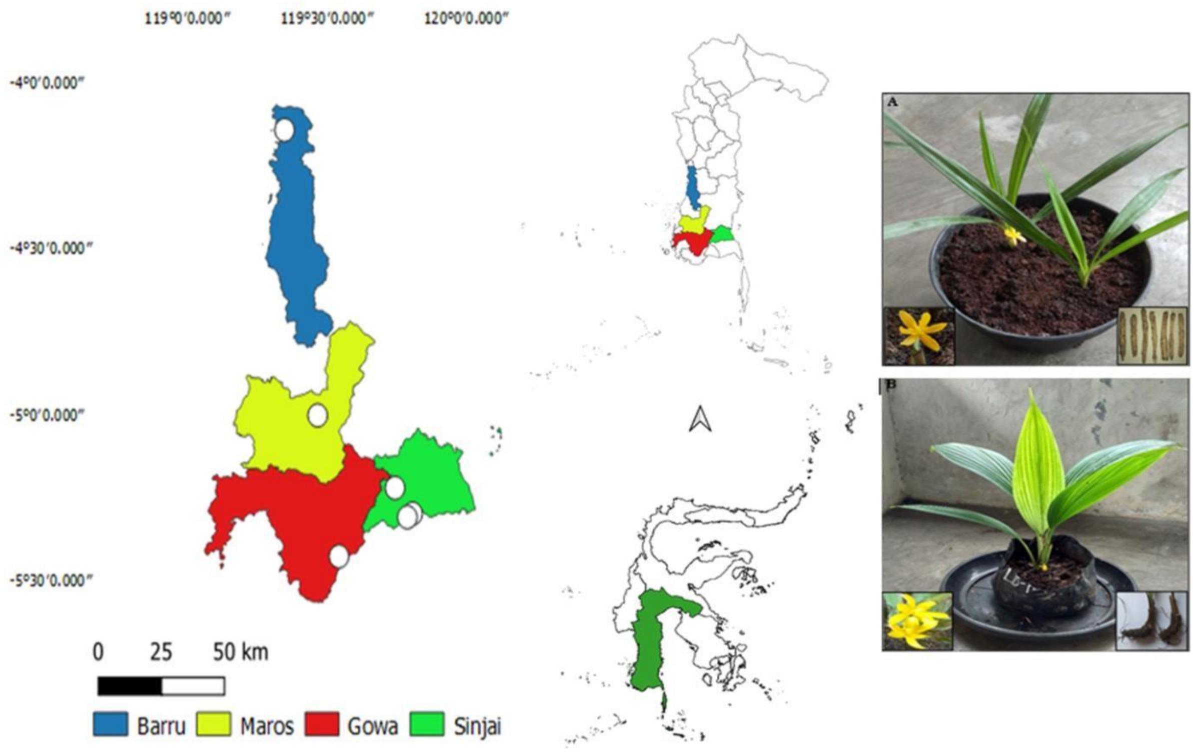

Figure S7. Sampling location of *Curculigo* spp. in South Sulawesi, Sulawesi Island, Indonesia. *Curculigo orchioides* Gaertn. (**A**) and *Curculigo latifolia* Dryand. ex W.T.Aiton (**B**).
